# Important Structural Features of Thiolate-Rich Four-Helix Bundles for Cu(I) Uptake and Removal

**DOI:** 10.1021/acs.inorgchem.2c04490

**Published:** 2023-04-14

**Authors:** Jaeick Lee, Rosemary A. Dalton, Arnaud Baslé, Nicolas Vita, Christopher Dennison

**Affiliations:** Biosciences Institute, Newcastle University, Newcastle upon Tyne NE2 4HH, U.K.

## Abstract

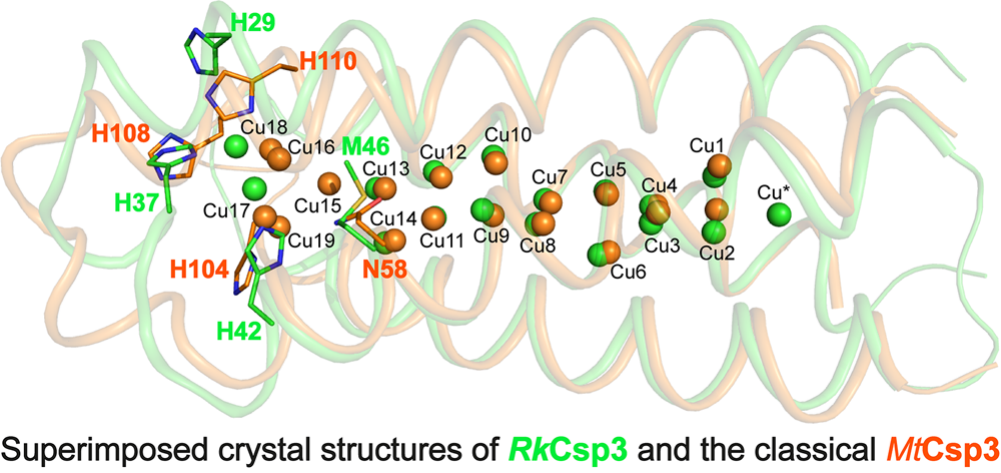

A family of bacterial copper storage proteins (the Csps) possess thiolate-lined four-helix bundles whose cores can be filled with Cu(I) ions. The majority of Csps are cytosolic (Csp3s), and in vitro studies carried out to date indicate that the Csp3s from *Methylosinus trichosporium* OB3b (*Mt*Csp3), *Bacillus subtilis* (*Bs*Csp3), and *Streptomyces lividans* (*Sl*Csp3) are alike. Bioinformatics have highlighted homologues with potentially different Cu(I)-binding properties from these characterized “classical” Csp3s. Determination herein of the crystal structure of the protein (*Rk*Csp3) from the methanotroph *Methylocystis* sp. strain Rockwell with Cu(I) bound identifies this as the first studied example of a new subgroup of Csp3s. The most significant structural difference from classical Csp3s is the presence of only two Cu(I) sites at the mouth of the bundle via which Cu(I) ions enter and leave. This is due to the absence of three Cys residues and a His-containing motif, which allow classical Csp3s to bind five to six Cu(I) ions in this region. Regardless, *Rk*Csp3 exhibits rapid Cu(I) binding and the fastest measured Cu(I) removal rate for a Csp3 when using high-affinity ligands as surrogate partners. New experiments on classical Csp3s demonstrate that their His-containing motif is not essential for fast Cu(I) uptake and removal. Other structural features that could be important for these functionally relevant in vitro properties are discussed.

## Abstract 2

Structural features influencing the functionality of cytosolic four-helix bundle copper storage proteins (Csp3s) have been investigated. The crystal structure of a new Csp3 subfamily member defines how sequence variations can influence the number and coordination of Cu(I) sites. The impact of the presence and location of His ligands around the mouth of the bundle and a coordinating residue at the interface between the mouth and the main core on Cu(I) binding and removal is discussed.

## Introduction

Copper (Cu) is required by both prokaryotes and eukaryotes for a range of proteins, primarily due to its facile redox chemistry.^1−6^ However, this reactivity, and the ability of intracellular Cu(I) to bind tightly to sites for other metals in proteins, results in potential dangers associated with the use of this metal.^3−9^ Homeostasis systems involving an array of proteins and some small molecules help cells safely handle Cu,^2−6,10−12^ including cytosolic storage by metallothioneins (MTs), mainly in eukaryotes. A family of proteins that can store Cu in bacteria, the Csps, was first identified in the methanotroph *Methylosinus trichosporium* OB3b (*Mt*).^13^ The predicted twin-arginine translocase (Tat) exported *Mt*Csp1 stores Cu(I) for the membrane-bound (particulate) methane monooxygenase (pMMO). A cytosolic homologue from the same methanotroph (*Mt*Csp3) has been characterized in vitro,^14,15^ but its function remains to be determined. Using *Escherichia coli* as a heterologous in vivo model system has shown that *Mt*Csp3, and the protein from *Bacillus subtilis* (*Bs*Csp3), can safely store Cu(I) in the cytosol and prevent the toxicity caused by increased intracellular Cu concentrations.^16^ Recent work has demonstrated that *Bs*Csp3 stores Cu(I) for the endospore multicopper oxidase CotA,^17^ the only known function for a Csp3.

Csps are tetramers of four-helix bundles,^13−15,18,19^ a common fold for proteins that naturally bind metals (metalloproteins) and one that is also widely used in metal-site design.^19−26^ A highly unusual feature of the Csps is the large number of Cys residues they possess, which all point into the center of the bundle and do not form disulfide bonds. This allows the proteins to be filled with Cu(I) ions bound predominantly by Cys thiolates, giving an arrangement of metal sites unlike that seen in any other metalloprotein. This way of storing Cu(I) is therefore very different from how the unstructured Cys-rich apo-MTs sequester cuprous ions by folding around two Cu(I) clusters.^19,27−29^ There is limited information about the mechanisms of Cu(I) uptake by, and removal from, Csps.^15,18,19^ The kinetics of these processes, along with how tightly Csps bind Cu(I), studied primarily using Cu(I) ligands, are key in vitro characteristics that have been measured to help understand how homeostasis proteins safely bind, store, and chaperone Cu(I) in cells.^13,14,18,29−41^

**Figure 1:**
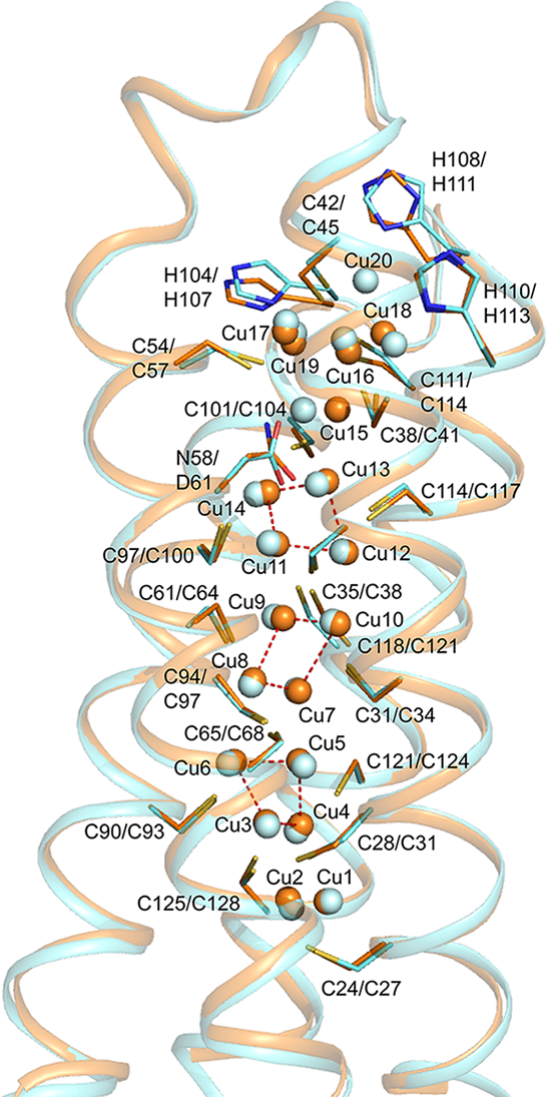
Comparison of currently available Cu(I)-Csp3 crystal structures. An overlay of the classical Cu(I)-*Mt*Csp3 (orange)^14^ and Cu(I)-*Sl*Csp3 (cyan)^42^ structures demonstrating their similarity [the root-mean-square deviation (rmsd) for C^α^ atoms is 0.41 Å]. In particular, most Cu(I) sites are superimposable. Coordinating side chains are represented as sticks, and Cu(I) ions as spheres. Strong Cu(I) to Cu(I) interactions are shown with dashed red lines for Cu(I)-*Mt*Csp3, and the numbering of Cu(I) sites is based on this structure (the first reported),^14^ with side chains labeled for both Cu(I)-*Mt*Csp3 and Cu(I)-*Sl*Csp3 in this order.

Crystal structures are available for the fully Cu(I)-loaded forms of *Mt*Csp3 and the protein (*Sl*Csp3) from *Streptomyces lividans* (Figure 1).^14,42^ The structure of apo-*Bs*Csp3 has also been determined.^14^ In both Cu(I)-*Mt*Csp3 and Cu(I)-*Sl*Csp3, the main Cu(I) core is made up of three thiolate-coordinated tetranuclear clusters.^15^ A number of Cu(I) sites are located closer to the mouth of the bundle and involve coordination from three His residues in the α3-loop-α4 region as well as five Cys residues.^14,42^ This His-containing motif is present in *Mt*Csp3, *Sl*Csp3, and *Bs*Csp3 and many other Csp3s (Figure S1 and refs (14) and (18)). As these were the first characterized members of this family of proteins, we refer to them as “classical” Csp3s. Homologues predicted to have the same Cu(I) core structure but with Cu(I)-binding differences elsewhere in the bundle have been identified from sequence alignments (Figure S1). In particular, the absence of three Cys residues (Cys38, Cys54, and Cys111, *Mt*Csp3 numbering) and the α3-loop-α4 His-containing motif suggest dramatic alterations in the Cu(I) sites bound at the mouth of the bundle. In this work, a nonclassical Csp3 has been analyzed in detail, including determination of its crystal structure fully loaded with Cu(I). Comparisons to classical Csp3s are assisted by new data for *Mt*Csp3, *Sl*Csp3, and *Bs*Csp3.

## Results and Discussion

### Cu(I) Binding Stoichiometries and the Average Cu(I) Affinities of Csp3s

**Figure 2:**
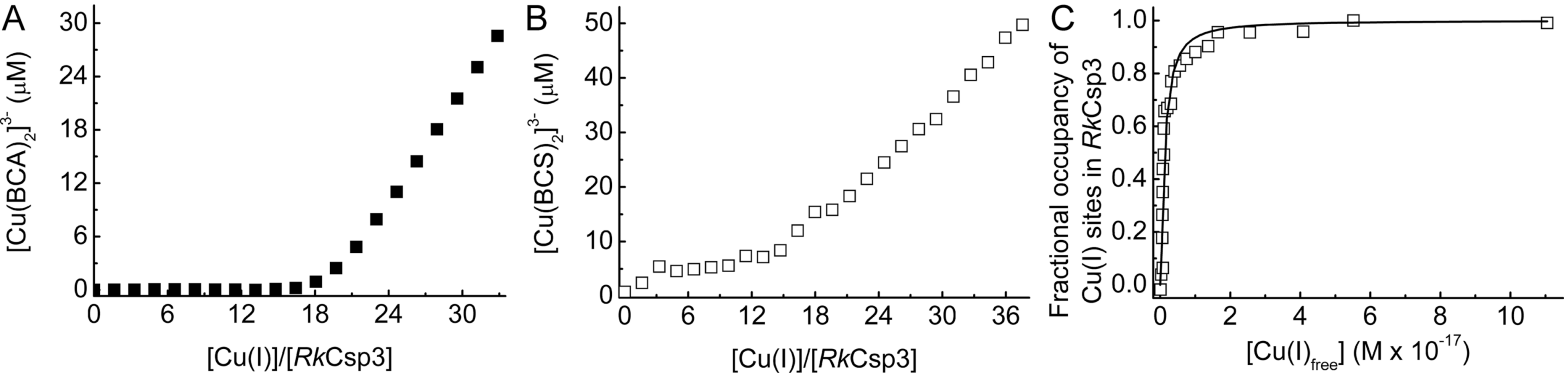
Cu(I) binding by *Rk*Csp3. (A) A plot of [Cu(BCA)_2_]^3–^ concentration against the [Cu(I)]/[*Rk*Csp3] ratio for a monomer (2.54 μM) in the presence of 106 μM bicinchoninic acid (BCA) giving 18.9 equiv of Cu(I) bound to the protein. (B) A plot of [Cu(BCS)_2_]^3–^ concentration against the [Cu(I)]/[*Rk*Csp3] ratio for mixtures of *Rk*Csp3 (2.51 μM) and Cu(I) in the presence of 121 μM bathocuproine disulfonic acid (BCS) incubated for 96 h. (C) A plot of the fractional occupancy of Cu(I) sites in *Rk*Csp3 against the free Cu(I) concentration ([Cu(I)_free_]),^13,14^ calculated from the data in (B). The maximum calculated occupancy is 17.3 Cu(I) equiv per monomer, with experimental values of 19.5 [determined using BCS to quantify Cu(I)] and 18.1 [using the Cu concentration measured by atomic absorption spectroscopy (AAS)] for samples at the highest added Cu(I) concentrations. The solid line in (C) shows a fit of the data to the nonlinear Hill equation giving an average dissociation constant for Cu(I), *K*_Cu_, of (0.12 ± 0.01) × 10^–17^ M and Hill coefficient of 1.3 ± 0.2. All experiments were carried out in 20 mM 4-(2-hydroxyethyl)-1-piperazineethanesulfonic acid (HEPES) pH 7.5 plus 200 mM NaCl.

Sequence comparisons predict a large subgroup of Csp3s (Figure S1), with properties different to those of classical homologues.^14^ To test this prediction, the Csp3 from the methanotroph *Methylocystis* sp. strain Rockwell (*Rk*Csp3) has been studied. The Cu(I)-binding capacity of *Rk*Csp3 (Figure 2A, Figure S2 and the Supporting Results) is similar to those for classical Csp3s (Table 1, Figures S3 and S4, the Supporting Results, and refs (14) and (42)), with a calculated stoichiometry of 18.0 ± (standard deviation) 0.6 (*n* = 5) mol equiv of Cu(I) per monomer. The average Cu(I) affinity of *Rk*Csp3 is (8.2 ± 1.1) × 10^17^ M^–1^ (Figure 2B,C, *n* = 3). This is approximately 5-fold tighter than those of *Mt*Csp3,^14^*Bs*Csp3,^14^ and *Sl*Csp3 (Table 1, Figure S5, and the Supporting Results), which all have similar average Cu(I) affinities [(1–2) × 10^17^ M^–1^]. The Hill coefficient for Cu(I) binding to *Rk*Csp3 (1.7 ± 0.5, *n* = 3) indicates slight positive cooperativity, while the values for *Mt*Csp3, *Bs*Csp3, and *Sl*Csp3 (Table 1, Figure S5, and the Supporting Results) are all ∼1.

**Table 1:** Comparison of Lengths, Number of Key Amino Acid Residues, Secondary and Quaternary Structures, and Cu(I)-Binding Features of Csp3s

	protein			
property	*Rk*Csp3	*Mt*Csp3	*Sl*Csp3^a^	*Bs*Csp3
length (amino acids)^b^	115	133	136	108
Cys residues^b^	16	18	18	19
His residues^b^	6	7	4	6
α-helical content of the apo-protein^c^	67.6 ± 2.5% (*n* = 3)	76.0 ± 2.8% (*n* = 10)^14^	85.9 ± 1.3% (*n* = 3)	83.1 ± 2.5% (*n* = 3)^14^
α-helical content of the Cu(I) protein^c^	66.1 ± 1.1% (*n* = 3)	74.8 ± 2.2% (*n* = 4)^14^	83.4 ± 1.2% (*n* = 3)	76.0 ± 2.6% (*n* = 3)^14^
apparent *M*_r_ of the apo-protein^d^	45 ± 1 kDa (*n* = 3)	45 ± 4 kDa (*n* = 31)^14^	55 ± 1 kDa (*n* = 3)	46 ± 2 kDa (*n* = 8)^14^
apparent *M*_r_ of the Cu(I) protein^d^	44 ± 1 kDa (*n* = 3)	45 ± 2 kDa (*n* = 12)^14^	50 ± 1 kDa (*n* = 3)	43 ± 1 kDa (*n* = 8)^14^
Cu(I) binding stoichiometry^e^	18.0 ± 0.6 (*n* = 5)	17.9 ± 1.0 (*n* = 8)^14^	18.2 ± 0.5 (*n* = 5)	19.6 ± 0.8 (*n* = 5)^14^
average Cu(I) affinity^f^	(8.2 ± 1.1) × 10^17^ M^–1^ (*n* = 3)	(1.7 ± 0.5) × 10^17^ M^–1^ (*n* = 3)^14^	(1.6 ± 0.1) × 10^17^ M^–1^ (*n* = 3)	(1.5 ± 0.4) × 10^17^ M^–1^ (*n* = 3)^14^
Hill coefficient^f^	1.7 ± 0.5 (*n* = 3)	1.0 ± 0.1 (*n* = 5)^14^	1.0 ± 0.1 (*n* = 3)	0.9 ± 0.1 (*n* = 3)^14^

a *Sl*Csp3 has been studied previously^42^ with our analysis carried out only to enable a direct comparison to data for the other Csp3s we have characterized (see the Supporting Results for more information).

b Sequence alignments are provided in Figure S1.

c Determined using the mean residue ellipticity at 222 nm obtained from far-UV circular dichroism (CD) spectra.^14^

d Determined in solution using analytical gel-filtration chromatography.^13,14^

e Calculated from experiments in the presence of an approximately 20-fold excess of BCA (the quoted values are per monomer).

f Average Cu(I) affinities measured using a protein concentration of ∼2.5 μM and BCS (∼120 μM) to buffer the concentration of available Cu(I) with data fit to the nonlinear Hill equation. Where relevant, averages and standard deviations are shown for a number of repeats (*n* values) of an experiment.

### Crystal Structure of Cu(I)-*Rk*Csp3 Compared with Those of Cu(I)-*Mt*Csp3 and Cu(I)-*Sl*Csp3

**Figure 3:**
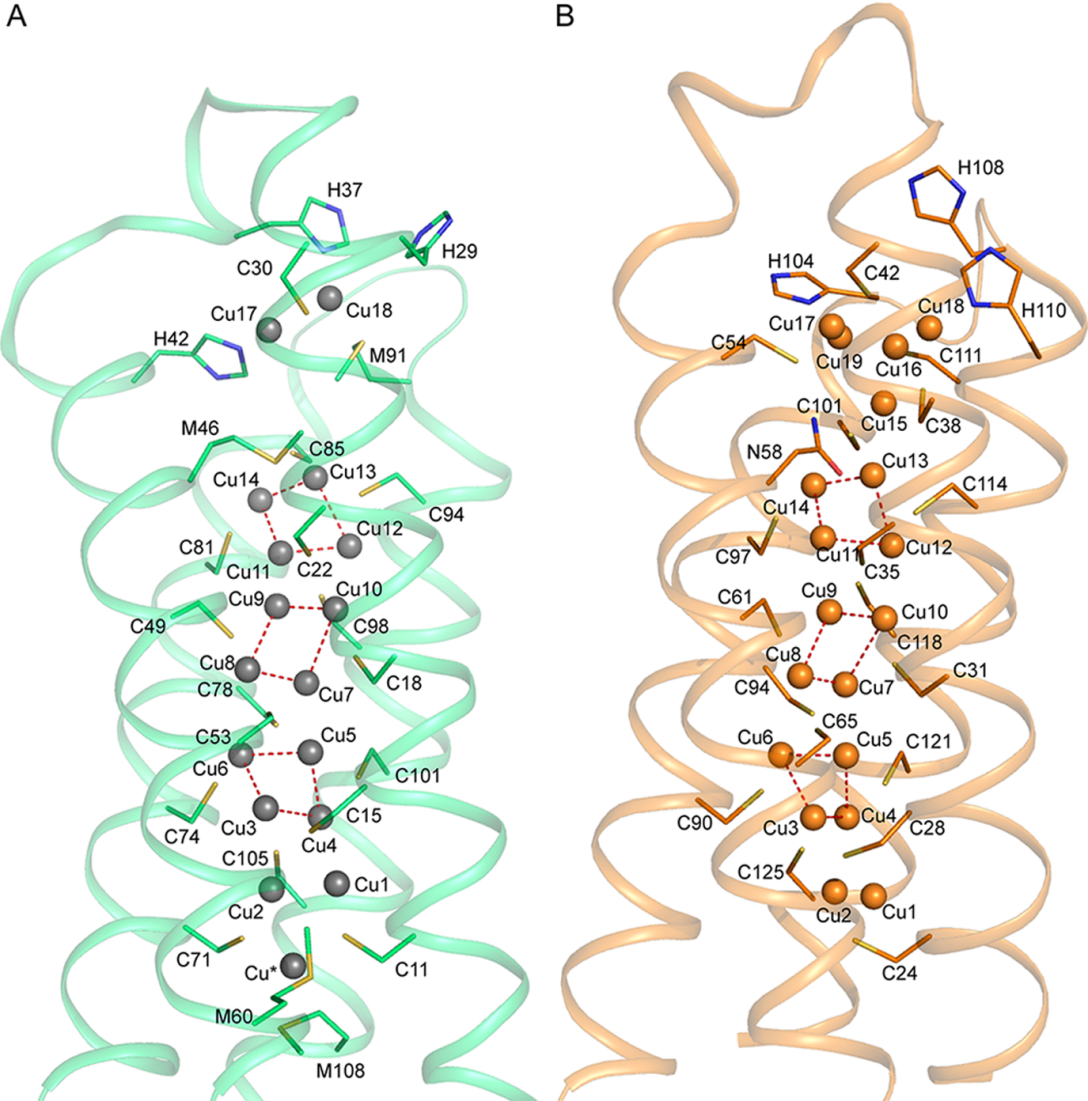
Comparison of Cu(I)-Csp3 structures. (A) The arrangement of the Cu(I) ions within the core of the four-helix bundle of the crystal structure of Cu(I)-*Rk*Csp3 (green). For comparison, the structure of the classical Cu(I)-*Mt*Csp3 (orange)^14^ is shown in (B). Coordinating side chains are represented as sticks, and Cu(I) ions as dark-gray and orange spheres. Strong Cu(I) to Cu(I) interactions are shown as in Figure 1, and the numbering of Cu(I) sites in *Rk*Csp3 is based on that for the crystal structure of Cu(I)-*Mt*Csp3 (Table 2).^14^

To gain more detailed information about Cu(I) binding by *Rk*Csp3, the crystal structure of the protein fully loaded with Cu(I) has been determined. There are Cu(I) ions bound at 17 sites (Figure 3A, Table 2, and Figure S6), consistent with the above stoichiometry studies. The protein has a helical content of ∼70%, which agrees with far-UV CD data (Table 1 and Figure S7A) and is unaffected by Cu(I) binding, as is the case for other Csps (Table 1, Figure S8A, and refs (13) and (14)). Cu(I)-*Rk*Csp3 forms a tetrameric arrangement in crystals (Figure S6), and both the Cu(I)- and apo-proteins are tetramers in solution (Table 1 and Figure S7B), as found for other Csps (Table 1, Figure S8B, Supporting Results, and refs (13), (14), and (42)). The structure of a Cu(I)-*Rk*Csp3 monomer is similar (rmsd for C^α^ atoms of 0.80 Å) to that of the unpublished structure of the apo-Csp3 (*Nm*Csp3) from *Nitrosospira multiformis* ATCC 25196 (3LMF) (Figure S9), which belongs to the same subgroup and whose sequence is similar (∼45% identity) to *Rk*Csp3 (Figure S1).

**Table 2:**
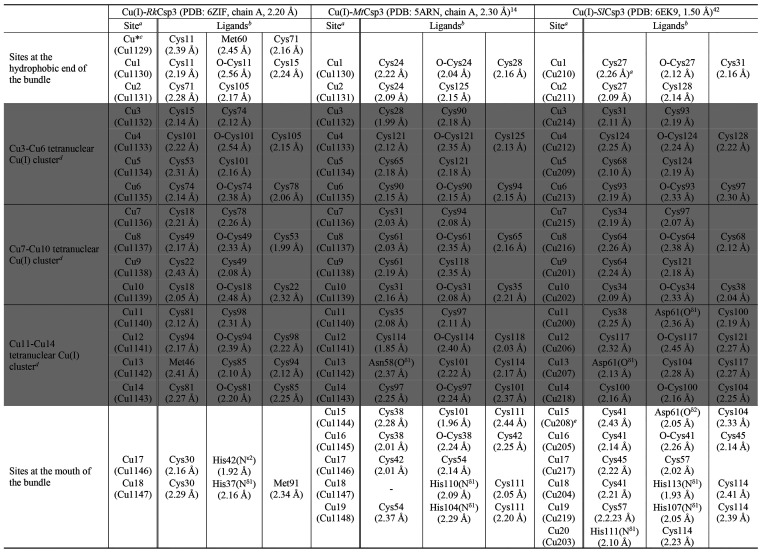
Comparison of Cu(I) Sites in the Crystal Structures of Cu(I)-*Rk*Csp3, Cu(I)-*Mt*Csp3, and Cu(I)-*Sl*Csp3

a The Cu(I) site numbering in parenthesis is as in the PDB files.

b Coordination is via a sulfur atom (thiolate of Cys or thioether of Met) unless stated otherwise.

c The thioether sulfur of Met108 is 2.59 Å from Cu* and may coordinate.

d These three clusters (highlighted grey) make up the main Cu(I) core.

e The thiolate sulfur of Cys57 is 2.60 Å from Cu15 in Cu(I)-*Sl*Csp3

**Figure 4:**
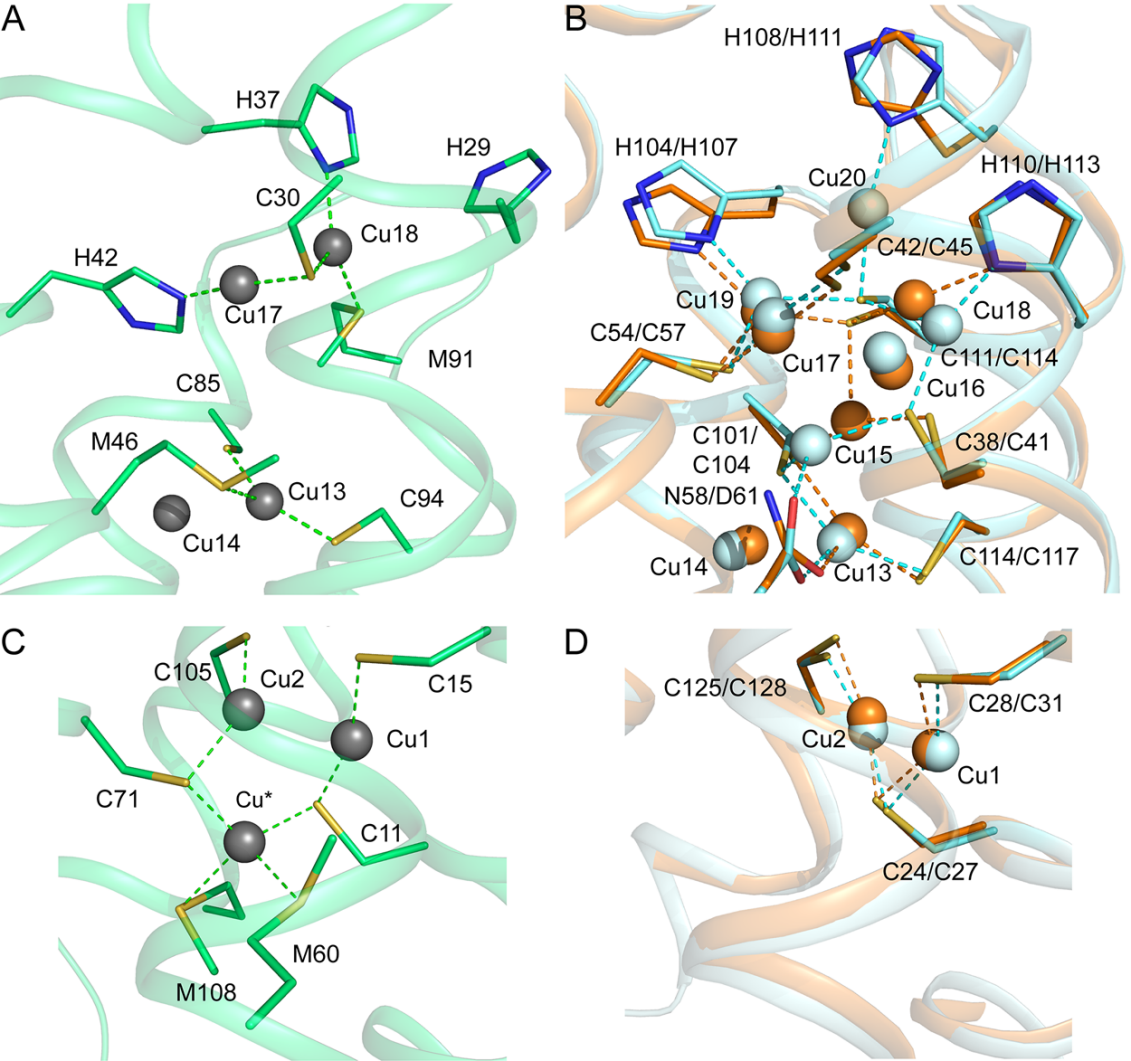
Detailed comparison of Cu(I) sites at the ends of the four-helix bundles of Csp3s. The sites at the mouth of Cu(I)-*Rk*Csp3 (A) and the corresponding region in an overlay of Cu(I)-*Mt*Csp3 and Cu(I)-*Sl*Csp3 (B) are shown, as are sites at the hydrophobic end of the bundle in (C) and (D). The proteins are colored, and Cu(I) sites and coordinating residues (see also Table 2) labeled, as in Figures 1 and 3. Cu(I)-ligand bonds are shown in green, orange, and cyan for Cu(I)-*Rk*Csp3, Cu(I)-*Mt*Csp3, and Cu(I)-*Sl*Csp3, respectively.

Three thiolate-coordinated tetranuclear Cu(I) clusters form the main core of Cu(I)-*Rk*Csp3, which is also the case in Cu(I)-*Mt*Csp3 and Cu(I)-*Sl*Csp3 (Figures 1 and 3 and Table 2).^14,15,42^ This is due to conservation of the Cys residues that are ligands at these sites (Figure S1). The only non-Cys ligand at these three clusters varies, with the side-chain thioether sulfur of Met46 coordinating Cu13 (at the Cu11–Cu14 cluster) in Cu(I)-*Rk*Csp3 (Figures 3A and 4A, Table 2). In *Mt*Csp3, the corresponding Asn58 coordinates Cu13 via its side-chain amide oxygen atom (O^δ1^) (Figure 4B and Table 2).^14^ This residue is located at the interface between the main Cu(I) core and sites at the mouth of the bundle, and in *Sl*Csp3 the carboxylate of Asp61 (O^δ1^) ligates not only Cu13 and Cu11 at the same tetranuclear cluster but also Cu15 via its O^δ2^ atom (Figure 4B and Table 2).^42^

There are differences in how *Rk*Csp3 binds Cu(I) at the two extremes of the bundle compared to classical Csp3s (Figure 3 and Table 2). The hydrophobic end furthest from the mouth has an additional Cu(I) site (Cu*; Figure 4C and Table 2). This is due to the presence of Cys71 (Leu87/90 in *Mt*Csp3/*Sl*Csp3, see Figure S1), Met60 (Ala72/Leu75 in *Mt*Csp3/*Sl*Csp3), and Met108 (Ala128/Leu131 in *Mt*Csp3/*Sl*Csp3). The thiolate of Cys11 also coordinates Cu* and bridges to Cu1. The corresponding Cys24/27 in *Mt*Csp3/*Sl*Csp3 bridges Cu1 and Cu2 (Figure 4D), while Cys71 provides the second thiolate for Cu2 in Cu(I)-*Rk*Csp3 (Figure 4C).

Significantly larger differences are seen when comparing Cu(I) sites at the mouth of the Cu(I)-*Rk*Csp3 bundle with those in classical Cu(I)-Csp3s (Figure 4A,B and Table 2). Cu(I)-*Mt*Csp3 binds a pentanuclear Cu(I) cluster including coordination of Cu18 and Cu19 by His110 and His104, respectively, both from the H^104^AGNH^108^EH^110^ α3-loop-α4 motif. In the structure of Cu(I)-*Sl*Csp3, there is an additional Cu(I) site (Cu20) bound by the central His111 residue of its corresponding (H^107^AGMH^111^EH^113^) motif (Figure 4B and Table 2).^42^ There are only two Cu(I) sites at the mouth of Cu(I)-*Rk*Csp3, and their positions most closely match those of Cu17 and Cu18 in Cu(I)-*Mt*Csp3 (Figure 4A,B and Table 2). The α3-loop-α4 His-containing motif is missing, but three His residues (highly conserved in the subfamily, see Figure S1) originating from the α1-loop-α2 region are present at the mouth of Cu(I)-*Rk*Csp3. The N^δ1^ atom of His37 coordinates Cu18 with the His42 N^ε2^ atom binding Cu17 (Figure 4A), while His29 does not act as a ligand. The tautomeric form of the His42 imidazole (N^δ1^H) is unusual as in all published Cu(I)-Csp structures His residues exhibit N^δ1^ atom coordination (see Table 2 for Csp3s).^13−15,42^ The thiolate of the only Cys residue (Cys30) in this region of *Rk*Csp3 (there are four in *Mt*Csp3 and *Sl*Csp3; see Figure 4 and Table 2) bridges Cu17 and Cu18, with the latter also bound by Met91 (Cu17 is two-coordinate).

### Filling the Cores of Csp3s with Cu(I) Ions

The ability of *Rk*Csp3 and the three classical Csp3s; *Mt*Csp3, *Sl*Csp3, and *Bs*Csp3 to bind Cu(I) has been studied. The high-affinity chromophoric chelator bicinchoninic acid (BCA) was used as the surrogate partner (i.e., by monitoring the loss of Cu(I) from [Cu(BCA)_2_]^3–^), and data have been acquired over a range of [Cu(BCA)_2_]^3–^ concentrations. This approach is similar to that reported previously for studying the binding of Cu(I) to the initial sites occupied in *Sl*Csp3.^65^ The authors of this work state they have discrepancies between the amount of Cu(I) added and the uptake of Cu(I), which appears to become more significant at higher equiv. However, we have not experienced this issue with any of the Csp3s and have focused on reactions adding sufficient Cu(I) to approximately half and fully load the proteins.

**Figure 5:**
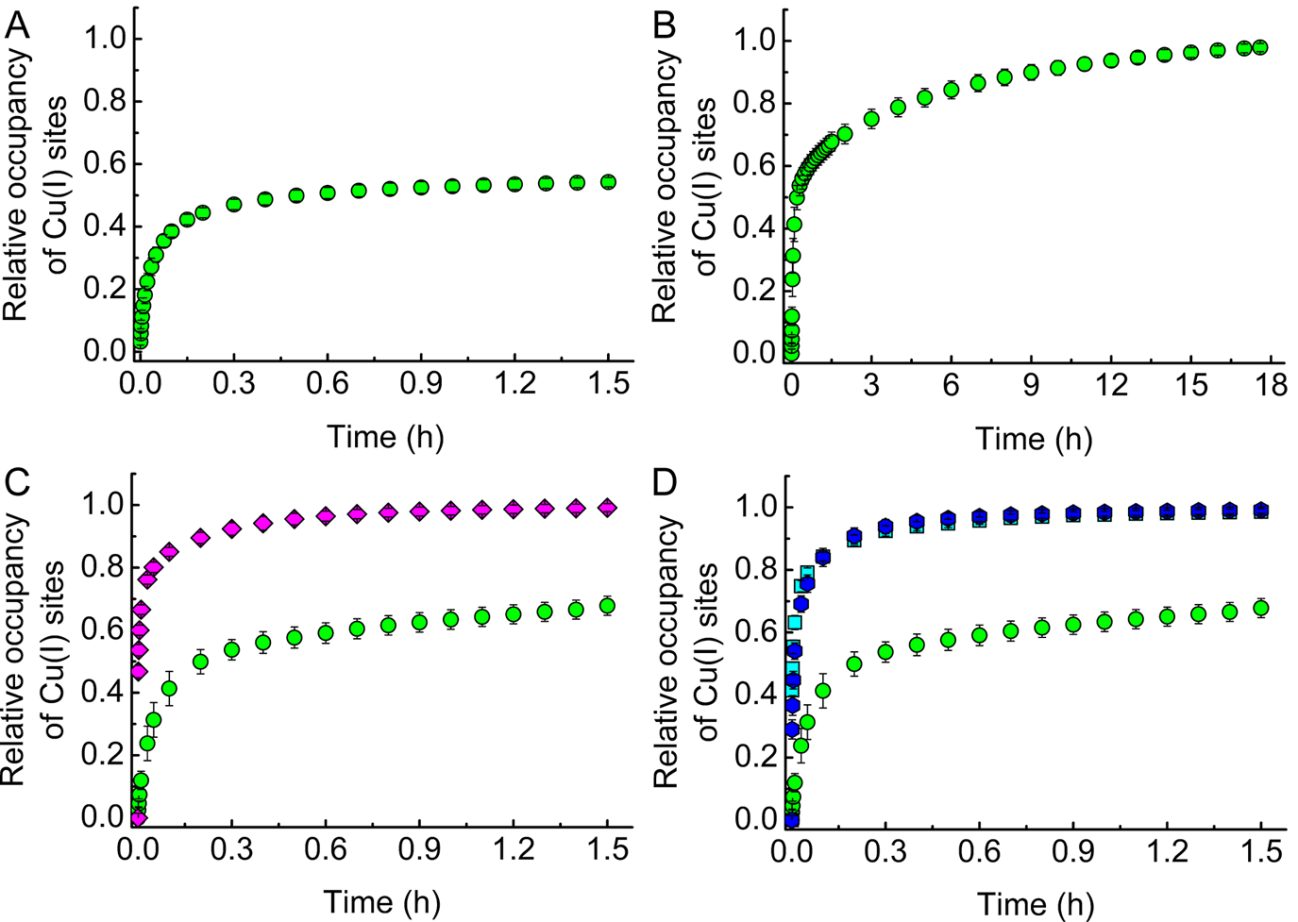
Filling the cores of Csp3s with Cu(I). Plots of relative occupancy (compared to the value at 24 h) for half (A) and full (B) loading of *Mt*Csp3 with Cu(I) from [Cu(BCA)_2_]^3–^, monitored for up to 1.5 and ∼18 h, respectively. The data in (B) up to 1.5 h are compared to full loading for *Rk*Csp3 (magenta diamonds) in (C) and *Sl*Csp3 (blue hexagons) and *Bs*Csp3 (cyan squares) in (D). All data were acquired in 20 mM HEPES pH 7.5 plus 200 mM NaCl at 22 °C, and plots show averages and errors as standard deviations. The experiments in (A) involved adding ∼10 equiv of [Cu(BCA)_2_]^3–^ to the apo-protein (∼2.1–2.4 μM), and a maximum occupancy of 10.0 ± 0.2 (*n* = 4) was measured. For full-loading (B–D), ∼17–19 equiv of [Cu(BCA)_2_]^3–^ was added to the apo-proteins (∼1.7–2.6 μM). Maximum occupancies of 16.1 ± 0.8 (*n* = 6), 16.8 ± 0.7 (*n* = 5), 16.9 ± 0.7 (*n* = 5), and 18.4 ± 0.4 (*n* = 4) were obtained for *Rk*Csp3, *Mt*Csp3, *Bs*Csp3, and *Sl*Csp3, respectively.

The analysis of the Cu(I)-binding data presented is based on experiments with *Mt*Csp3 (Figure 5A,B), as almost all of the half- and full-loading reactions are monitored for this protein. The kinetics of these reactions are multiphasic (see also ref (65)), and data have been fit to two and three exponentials, respectively (Figure S10). The rates obtained are listed in Table S1. The initial rate of Cu(I) uptake (*t*_1_) when half loading *Mt*Csp3 is ∼9-fold faster than the second step (*t*_2_). For full loading, very similar *t*_1_ and *t*_2_ values are obtained (Table S1), and a third stage is observed (*t*_3_) that is ∼15-fold slower than *t*_2_. In the crystal structure of *Mt*Csp3 plus ∼2 equiv of Cu(I), the only sites occupied, and presumably the most thermodynamically stable, are those at the tetranuclear cluster closest to the mouth of the bundle (Cu11–Cu14).^15^ Adding more Cu(I) results in further occupancy of this and the two other tetranuclear clusters (Cu3–Cu6 and Cu7–Cu10) that form the main Cu(I) core (Figures 1 and 3B and Table 2).^15^ The last sites to bind Cu(I) are those at the two extremes of the bundle, whose affinities must be weakest (access to sites at the hydrophobic end of the protein may be sterically hindered). The data therefore appear consistent with *t*_1_ and *t*_2_ being the rates for forming the main Cu(I) core, initially Cu11–Cu14. The slowest step (*t*_3_) corresponds to completing the process of filling *Mt*Csp3 with Cu(I), predominantly involving binding by sites at the hydrophobic end and the mouth of the bundle.

The core of *Rk*Csp3 fills much more quickly with Cu(I) ions from [Cu(BCA)_2_]^3–^ than does *Mt*Csp3, and the process is complete in <1 h (Figure 5C). Fitting data in the same way as described for *Mt*Csp3 gives the results shown in Table S1. Again, the two rates for half loading the protein with Cu(I) agree with the *t*_1_ and *t*_2_ values from fitting the full-loading experiments. We therefore assume these rates are also due to binding mainly at the three conserved tetranuclear clusters of the *Rk*Csp3 Cu(I) core (Figure 3 and Table 2),^14,42^ and both are ∼20-fold faster than for *Mt*Csp3 (Table S1). The last stage of filling the *Rk*Csp3 bundle with Cu(I) (*t*_3_) is ∼30-fold faster than in *Mt*Csp3. This process again probably involves Cu(I) binding largely to sites at the mouth and the hydrophobic end of the *Rk*Csp3 bundle (Figures 3A and 4A,C).

To further investigate the structural features of a Csp3 that are important for the rate of Cu(I) uptake, experiments were performed on two more classical Csp3s. *Sl*Csp3 and *Bs*Csp3 were chosen as reported in vitro data for these proteins (Table 1, Figures S3–S5 and S8, and ref (14)) closely match those for *Mt*Csp3.^14^ Half and full loading from [Cu(BCA)_2_]^3–^ were also fit in the same way as described for *Mt*Csp3. The rates for *Sl*Csp3 and *Bs*Csp3 (Table S1) were all significantly faster (*t*_1_ and *t*_2_ ≈ 20-fold and *t*_3_ ≈ 40-fold) than for *Mt*Csp3, and full loading is complete in <1 h for both proteins (Figure 5D), comparable to the data for *Rk*Csp3. We assume that the rates correspond to the same processes described above, particularly considering the high structural similarity of Cu(I)-*Mt*Csp3 and Cu(I)-*Sl*Csp3 (Figure 1). It should be noted that the initial Cu(I)-binding cluster in a crystal structure of *Sl*Csp3 is adjacent to that in *Mt*Csp,^15^ shifted towards the mouth of the bundle (Figure 1).^65^ However, these crystal structures were obtained at different pH values that could influence Cu(I) binding.

### Removing Cu(I) Ions from the Cores of Csp3s

**Figure 6:**
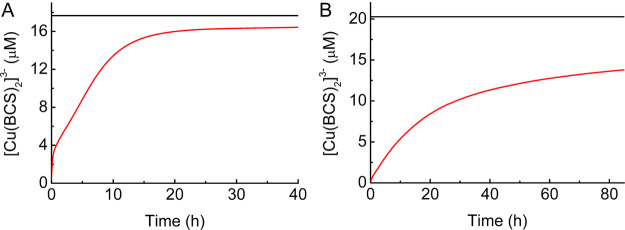
Cu(I) removal from Csp3s by BCS. The experiments shown were obtained when mixing Cu(I)-*Rk*Csp3 (1.08 μM) plus 15.9 equiv (A) and *Sl*Csp3 (1.11 μM) plus 18.1 equiv (B) of Cu(I) with ∼2.5 mM BCS. Data were measured in the absence (red line) and presence (black line showing the end point of the reaction) of 6.3 M guanidine hydrochloride in 20 mM HEPES pH 7.5 plus 200 mM NaCl at 22 °C.

The rates of Cu(I) removal from all four Csp3s have been studied using the higher-affinity chromophoric ligand bathocuproine disulfonic acid (BCS), i.e., by following the formation of the [Cu(BCS)_2_]^3–^ complex. This ligand was used rather than BCA as it acquires Cu(I) more rapidly from Csp3s. However, it still takes ∼20 h (Figure 6A) for all Cu(I) to be acquired from *Rk*Csp3 by BCS (∼2.5 mM). Removal of Cu(I) with BCS is slower for all classical Csp3s that have been tested, and only ∼12% is acquired from *Mt*Csp3 after 20 h (∼20% after 85 h).^14^ For *Sl*Csp3 (Figure 6B) and *Bs*Csp3,^14^ ∼70 and ∼80% of Cu(I) are removed after 85 h (∼40 and ∼50% at 20 h), respectively. Most Cu(I) removal data fit better to two exponentials (fits to one and two exponentials are similar for *Sl*Csp3), with an initial faster phase of low amplitude (Figure S11). The rates for the main (slower) phases are compared in Table S2. Because Cu(I) is removed slowly from *Mt*Csp3, the analysis of data for up to ∼85 h is unreliable, which is confirmed by the much smaller than expected changes in [Cu(BCS)_2_]^3–^ concentration obtained from fits. When the reaction was monitored for >600 h (>25 days), the rate was ∼10-fold slower (Table S2). Using this value, Cu(I) removal from *Rk*Csp3 is ∼100-fold faster than that from *Mt*Csp3, while the rates are ∼25-fold quicker for *Sl*Csp3 and *Bs*Csp3 (∼3.5-fold slower than *Rk*Csp3).

### Structural Features of Csp3s that Influence the Rates of Cu(I) Binding and Removal

Filling and emptying the core of *Rk*Csp3, the first characterized member of a new subgroup of Csp3s, via exchange with Cu(I) ligands are both relatively fast processes. The rates of Cu(I) uptake are similar to those for *Sl*Csp3 and *Bs*Csp3, but removal is slower in the classical Csp3s. For *Mt*Csp3 (another classical homologue), the Cu(I) uptake and removal rates are very slow compared to those of all other Csp3s studied here. Consequently, there appears to be no correlation between the structure at the mouth of the bundle (comparable in *Mt*Csp3, *Sl*Csp3, and *Bs*Csp3 but very different in *Rk*Csp3; see Figures 1, 3, and 4A,B, Table 2, and ref (14)) and the rates of Cu(I) binding and removal. This includes the influence of the number of Cu(I) sites in this region. Furthermore, the α3-loop-α4 His-ligand-containing motif, highly conserved in classical Csp3s, does not need to be present for these processes to be fast (*Rk*Csp3), and its presence can result in slow Cu(I) binding and removal (*Mt*Csp3). The lack of a specific role for these three His residues at the mouth of the *Mt*Csp3 and *Bs*Csp3 bundles for Cu(I) exchange with high-affinity ligands has been confirmed by making the H104A/H108A/H110A and H84A/H86A/H88A mutations, respectively. The former variant exhibits faster Cu(I) uptake (full loading is almost complete in ∼1.5 h) and removal (also finished in ∼1.5 h at pH 8.0) compared to the wild-type (WT) protein. Full loading with Cu(I) is not significantly influenced in the H84A/H86A/H88A-*Bs*Csp3 variant and complete Cu(I) removal occurs over a similar time scale to that of the WT protein (∼90% removal after 85 h), but the distribution of the two phases of the reaction changes. The study of single and double His mutants of *Sl*Csp3 have suggested the first His residue in the α3-loop-α4 motif (His107) is important for the initial entry of Cu(I) ions into this protein.^65^ The authors implicate a His107-Cu(I)-BCA intermediate in the mechanism of Cu(I) uptake. From our studies it appears that the formation of His-Cu(I)-BCA ternary complexes are not key for the reactions in which the cores of *Mt*Csp3 and *Bs*Csp3 are half and fully loaded with Cu(I) ions using BCA as a partner. In fact, these reactions are much faster in the absence of the α3-loop-α4 His residues in *Mt*Csp3. The presence of these His residues also does not enhance the rate of Cu(I) removal from Csp3s by BCS.

There are two His residues coordinating Cu17 and Cu18, the only Cu(I) sites at the mouth of Cu(I)-*Rk*Csp3, but these are present as different tautomers: His42 exists as the N^δ1^-H form (coordination via the N^ε2^ atom), with His37 in the N^ε2^-H form (Figure 4A). The coordination of a His via its N^ε2^ atom has not previously been observed in a Csp [the His residues in the α3-loop-α4 motif of classical Csp3s ligate via their N^δ1^ atoms (Figure 4B)].^13−15,42^ The significance of the tautomeric form of His ligands at biological Cu sites has been discussed for many years without any clear conclusions. His residues always coordinate mononuclear type 1 electron-transferring Cu sites via their N^δ1^ atoms, whereas N^ε2^ atom ligation is more common for type 2 and type 3 Cu sites in a range of enzymes.^1,20,21,43−46^ This is consistent with enhanced rates of catalysis for an engineered type 2 Cu site within a designed three-helix bundle scaffold when His residues coordinate via their N^ε2^ atoms.^47^ Information about His ligation in Csps is relatively limited, and it is too early to know if the coordination of Cu17 by the N^ε2^ atom of His42 in *Rk*Csp3 facilitates fast Cu(I) uptake and removal.

Another potential feature of Csp3s that could influence the rate of Cu(I) uptake and removal is the location of the first sites where Cu(I) ions bind within the core. In *Mt*Csp3, the sites forming the Cu11–Cu14 tetranuclear cluster (Figures 3B and 4B and Table 2) at which Asn58 is a ligand (to Cu13, Figure 4B) are the first to be occupied.^15^ Classical Csps typically have either an Asn or Asp residue at this position (Figure S1) located at the interface between the main Cu(I) core and sites at the mouth of the bundle, and the corresponding residue in *Sl*Csp3 is Asp61 (Asp42 in *Bs*Csp3) (Figures 1 and 4B, Table 2, and Figure S1). This coincides with faster rates of Cu(I) binding. Consistent with this observation, the Asn58Asp *Mt*Csp3 variant exhibits faster Cu(I) uptake than the WT protein (full loading occurs in ∼1.5 h), but this is not as fast as for *Sl*Csp3 and *Bs*Csp3. Whether the location of the initial Cu(I) cluster in *Mt*Csp3 (Cu11-Cu14)^15^ has been influenced by the Asn58Asp mutation, matching that in *Sl*Csp3,^65^ remains to be clarified. Cu(I) removal is less affected by the Asn58Asp mutation to *Mt*Csp3, with ∼30–40% acquired by BCS after 85 h (∼20% for the WT protein in the same time). A detailed analysis of Asn58Asp *Mt*Csp3 and other variants mentioned herein will be described in a subsequent paper. However, Asn58 is partially responsible for the slow Cu(I) uptake rates of *Mt*Csp3 and presumably other classical homologues with the same residue in this position. If the α3-loop-α4 motif is missing, as in the new subgroup of Csp3s identified in this work, then the residue corresponding to Asn58 of *Mt*Csp3 is usually a Met (Met46 in *Rk*Csp3, see Figure S1). This coincides with faster Cu(I) uptake and removal.

**Figure 7:**
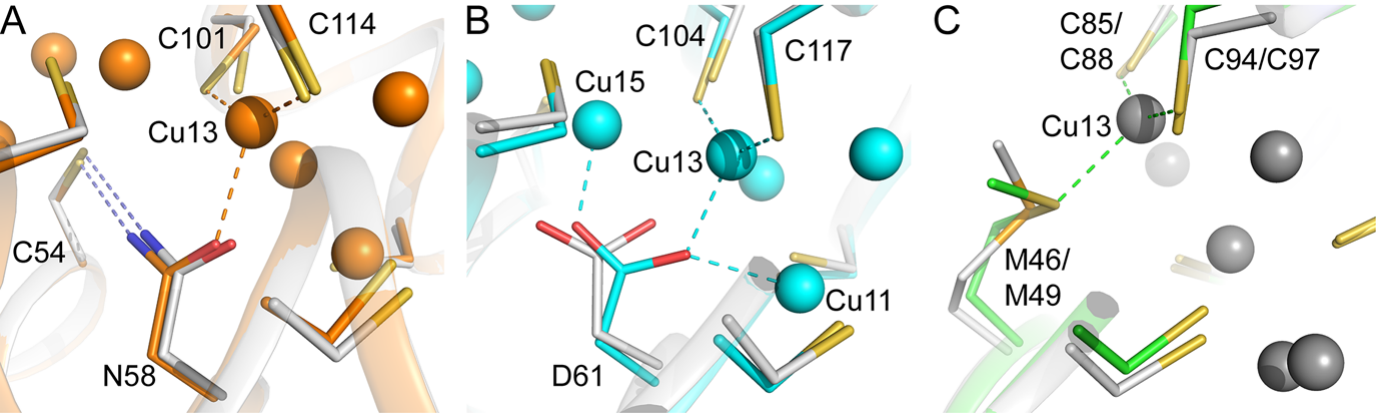
The arrangement around Asn58, Asp61, and Met46 in structures of *Mt*Csp3, *Sl*Csp3, and *Rk*Csp3, respectively. The orientations of the Asn58, Asp61, and Met46 side chains in the crystal structures of Cu(I)-*Mt*Csp3 (A),^14^ Cu(I)-*Sl*Csp3 (B),^42^ and Cu(I)-*Rk*Csp3 (C), colored as in Figures 1 and 3, are compared with those in the apo-proteins (apo-*Nm*Csp3 is used in C) that are all colored light gray. The side chains of Asn58, Asp61, and Met46 ligate Cu13 in *Mt*Csp3, *Sl*Csp3, and *Rk*Csp3, respectively, while Asp61 also coordinates Cu11 and Cu15 (Table 2). Cu(I)-ligand bonds are shown as dashed lines of the appropriate color, and one of the hydrogen bonds between Asn58 and Cys54 in the *Mt*Csp3 structures is shown as a slate dashed line in (A).

A possible reason that Asn58 slows Cu(I) uptake in *Mt*Csp3 is the hydrogen bonds made by its noncoordinating N^δ2^H_2_ group, including with the thiolate sulfur (Figure 7A) and backbone carbonyl oxygen of Cys54.^14^ These interactions fix the conformation of the Asn58 side chain, which is the same in the structures of Cu(I)- and apo-*Mt*Csp3 (Figure 7A).^14^ In *Sl*Csp3, such interactions are missing and the orientation of Asp61 is different in the apo- and Cu(I)-protein structures (Figure 7B) with this residue coordinating three different Cu(I) sites (Table 2). These observations are consistent with previous findings that a carboxylate side chain can readily exhibit changes in coordination at metal sites in proteins.^48,49^ The Met side chain is unbranched, flexible, and cannot be involved in hydrogen bonding. There appears to be space within the *Rk*Csp3 core for the side chain of Met46 to adopt different conformers, which is the case for Met49 in the apo-*Nm*Csp3 structure (Figure 7C). Conformational flexibility of the residue at the position corresponding to Asn58 of *Mt*Csp3 could be key for faster Cu(I) uptake.

## Conclusions

Sequence alignments indicated the presence of Csp3s with a different arrangement of Cu(I) sites at the mouth of their four-helix bundles compared to previously studied classical homologues (Figure S1). The crystal structure of the Cu(I)-form of such a protein determined herein, confirms this to be the case, and *Rk*Csp3 is the first characterized example of a new subgroup of Csp3s. These proteins possess only two Cu(I) sites at the mouth of their bundles, compared to five or six in classical Csp3s (Figures 3 and 4A,B). This is due to having only a single Cys residue (Cys30) in this region compared to four in classical Csp3s, and the absence of the α3-loop-α4 His-containing motif. *Rk*Csp3 has an additional Cu(I) site at the hydrophobic end of the bundle coordinated by two Met residues as well as two Cys thiolates. *Rk*Csp3 can bind up to 17 Cu(I) ions per monomer (Table 1), which is very similar to the Cu(I) capacities of *Mt*Csp3, *Sl*Csp3, and *Bs*Csp3 (∼18–20). The protein has an average Cu(I) affinity approximately 5-fold tighter than those of classical Csp3s (Table 1). The time taken to fully occupy the core of *Rk*Csp3 with Cu(I) from [Cu(BCA)_2_]^3–^ is similar to that of some classical Csp3s (*Sl*Csp3 and *Bs*Csp3), but this process is dramatically slower for *Mt*Csp3. The removal of Cu(I) from *Rk*Csp3 by BCS, although it still occurs over many hours, is the fastest observed to date for a Csp3. The arrangement of sites at the mouth of *Rk*Csp3, along with the flexible Met46 residue at the interface with the main core, appears to promote both faster Cu(I) uptake and removal. A Csp four-helix bundle provides two α-loop-α motifs at its mouth able to accommodate different structural features that can influence Cu(I) uptake and removal. However, the rates of these reactions, and particularly filling the cores of classical Csp3s with Cu(I), appear not to depend on the α3-loop-α4 His-containing motif. The presence of Asn58 in *Mt*Csp3, rather than the Asp residue at this key position in *Sl*Csp3 and *Bs*Csp3 (Met in *Rk*Csp3), contributes to Cu(I) uptake being very slow. The architecture at the mouth of a Csp3’s four-helix bundle is not the only important feature for physiologically relevant in vitro properties.

## Methods

### Cloning, Overexpression, and Purification of Proteins

The *csp3* gene from *Methylocystis sp.* ATCC 49242 (Rockwell) was amplified from genomic DNA using the following primers; 5′-GGAGGACGC**CATATG**CACAAAATGTCCAAGG-3′ (forward, *Nde*I restriction site in bold) and 5′-CGCAAGCGC**CCATGG**TCACGCCGCCATCTTTTGTCCC-3′ (reverse, *Nco*I restriction site in bold, stop codon underlined). The fragment was ligated into pGEM-T (Promega), verified by sequencing, and subcloned into the *Nde*I and *Nco*I sites of pET29a (this was carried out in two steps as the gene contains an *Nde*I site) to give pET29a_*Rk*Csp3. The *Sl*Csp3gene was synthesized with codons optimized for expression in *Escherichia coli* (GENEWIZ) and subcloned into the *Nde*I and *Hind*III sites of pET29a, giving pET29a_*Sl*Csp3.

*E. coli* BL21 (DE3) transformed with either pET29a_*Rk*Csp3 or pET29a_*Sl*Csp3 was grown in LB media at 37 °C (50 μg/mL kanamycin) until an OD_600_ of ∼0.5 to 0.8 was achieved. Cells were induced with 0.1 and 1 mM isopropyl β-d-1-thiogalactopyranoside, grown for 24 h, and harvested by centrifugation at 5000*g* for 10 min, and pellets were stored at −30 °C. *Sl*Csp3 was purified using the same procedure as for *Mt*Csp3.^13,14^ Purification was more complex for *Rk*Csp3 due to relatively weak binding to ion-exchange columns. The first step typically involved using a HiTrap Q HP column (5 mL, sometimes two columns connected) equilibrated in 2–20 mM tris(hydroxymethyl)aminomethane (Tris) pH 8.5 plus 1 mM dithiotheritol (DTT) and eluted with a linear NaCl gradient of ∼0–500 mM. The *Rk*Csp3-containing fractions, identified by 18% sodium dodecyl sulfate polyacrylamide gel electrophoresis (SDS-PAGE), were further purified on a HiTrap SP HP column (5 mL) in 1–10 mM 2-(*N*-morpholino) ethanesulfonic acid pH 6.0 plus 1 mM DTT and eluted with a linear NaCl gradient (∼0 up to ∼500 mM). Regardless of the buffer concentration, some protein did not bind to this column, and further purification of the flow-through and eluted fractions was carried out in 10–20 mM Tris pH 7.0 to 8.5 plus 1 mM DTT on either a HiTrap Q HP column (5 mL) or connected HiTrap Q and HiTrap SP HP columns (5 mL). When anion and cation exchange columns were used in series, the two columns were eluted separately using ∼0–400 mM NaCl gradients, and fractions from the anion-exchange column were further purified on a HiTrap SP HP column in 20 mM HEPES pH 7.0 plus 1 mM DTT. The purest fractions, as determined by SDS-PAGE, were concentrated and exchanged into 20 mM HEPES pH 7.5 plus 200 mM NaCl by ultrafiltration [Amicon stirred cell with a 10 kDa molecular weight cutoff (MWCO) membrane]. The final purification step utilized a Superdex S75 10/300 GL gel-filtration column, equilibrated in 20 mM HEPES pH 7.5 plus 200 mM NaCl.

Purified *Rk*Csp3 and *Sl*Csp3 were analyzed for Cu and Zn(II) by AAS as described previously.^13,14^ Both proteins contained a negligible amount of Cu (≤0.1 equiv) and a maximum of ∼0.4 equiv of Zn(II). The molecular masses of proteins were as expected when measured using liquid chromatography–mass spectrometry.

### Purification of *Mt*Csp3 and *Bs*Csp3

*Mt*Csp3 and *Bs*Csp3 were purified using previously reported protocols.^13,14^

### Protein Quantification

All procedures, apart from Bradford assays (see below), were carried out under strict anaerobic conditions using an anaerobic chamber (Belle Technology, [O_2_] ≪2 ppm), gastight syringes (Hamilton), and sealable quartz cuvettes (Hellma). UV/vis spectra were recorded on a λ35 spectrophotometer (PerkinElmer). The concentration of apo-Csp3s was determined using the 5,5′-dithiobis(2-nitrobenzoic acid) (DTNB, Ellman’s reagent) assay to quantify thiols,^50^ under anaerobic conditions as described previously.^13,14^ These measurements assumed 16 and 18 free thiols for *Rk*Csp3 and *Sl*Csp3, respectively. For all of the Cys residues to be accessible in this assay, the proteins are unfolded, which was achieved in 20 mM HEPES pH 7.5 plus 200 mM NaCl containing 6.8 M guanidine hydrochloride (sometimes 8 M urea) and 1 mM ethylenediaminetetraacetic acid. The apo-Csp3s were also quantified using the Bradford assay with the Coomassie Plus protein assay kit (Thermo Scientific) as described previously.^13,14^ Bradford concentrations were divided by those obtained from the DTNB assay giving Bradford:DTNB ratios of 1.72 ± 0.22 (*n* = 28) and 1.18 ± 0.08 (*n* = 18) for *Rk*Csp3 and *Sl*Csp3, respectively. *Mt*Csp3 and *Bs*Csp3 were quantified in >7.0 M urea as described previously,^13,14^ giving the expected Bradford:DTNB ratios.

### Investigating Cu(I) Binding

A Cu(I) stock solution (typically 50 mM) prepared using [Cu(CH_3_CN)_4_]PF_6_ (Merck) in 100% anhydrous acetonitrile was diluted into 20 mM HEPES pH 7.5 plus 200 mM NaCl.^13,14,38,51^ The Cu(I) concentration of this working stock was determined with BCS that forms the [Cu(BCS)_2_]^3–^ complex with an ε value of 12 500 M^–1^ cm^–1^ at 483 nm.^38,52^ This was routinely compared to the total Cu concentration quantified using AAS. To monitor the effect of Cu(I) binding to proteins, the buffered Cu(I) solution was titrated into apo-Csp3s (typically ∼5 μM) and the formation of S(Cys)→Cu(I) ligand-to-metal charge-transfer bands was observed by UV/vis spectroscopy. Fluorescence was also measured during Cu(I) titrations on a Cary Eclipse fluorimeter (Varian), exciting at 280 nm and monitoring the emission in the 400–700 nm range with excitation and emission slits set to 10 and 20 nm, respectively.^13,51^ Cu(I) was also added to protein (∼2.5 μM) plus ∼100 μM BCA, which forms the [Cu(BCA)_2_]^3–^ complex with an ε value of 7700 M^–1^ cm^–1^ at 562 nm.^32,40,52^ This experiment was performed as either a titration for *Rk*Csp3 [equilibration for each Cu(I) addition is complete in ∼15 min] or by setting up a series of mixtures that were incubated and measured for up to 48 h for *Sl*Csp3 (slower equilibration).

### Investigating Cu(I) Uptake from the [Cu(BCA)_2_]^3–^ Complex

Cu(I) uptake from [Cu(BCA)_2_]^3–^ was investigated for *Rk*Csp3, *Sl*Csp3, *Mt*Csp3, and *Bs*Csp3. A stock solution of BCA (∼200 μM) in 20 mM HEPES pH 7.5 plus 200 mM NaCl was prepared in the anaerobic chamber, and the BCA concentration was determined by titration with a quantified Cu(I) stock solution. [Cu(BCA)_2_]^3–^ solutions (1 mL) at concentrations of typically 6.25, 12.5, 25.0, 37.5, and 50.0 μM were prepared in the anaerobic chamber. Apo-Csp3s (final concentration ∼1.7–2.6 μM) were rapidly added from a gastight syringe to a [Cu(BCA)_2_]^3–^ solution in a sealable cuvette, quickly mixed, and placed in a UV/vis spectrophotometer. The decrease in absorbance due to the removal of Cu(I) from the [Cu(BCA)_2_]^3–^ complex was monitored at 562 nm for up to 24 h at 22 °C. Plots of [Cu(BCA)_2_]^3–^ concentration against time were fit to a three phase model for Cu(I) binding using Origin 7. This involved fitting data from experiments adding sufficient [Cu(BCA)_2_]^3–^ to approximately half load the protein to two exponentials providing rates for the first two stages of uptake (*t*_1_ and *t*_2_). The full-loading data were fit to three exponentials that also provided the rate for the slowest stage of Cu(I) uptake (*t*_3_) as well as *t*_1_ and *t*_2_.

### Far-UV CD Spectroscopy and Analytical Gel-Filtration Chromatography

For far-UV (180–250 nm) CD spectroscopy, Cu(I)-Csp3s binding ∼17 to 18 mol equiv of Cu(I) was prepared by the addition of buffered Cu(I) (∼2–5 mM) to apo-protein (∼55 μM apo-*Rk*Csp3 and 138 μM apo-*Sl*Csp3) in 20 mM HEPES pH 7.5 plus 200 mM NaCl.^13,14^ Both the apo- and Cu(I)-proteins were transferred into either 20 or 100 mM phosphate pH 8.0 by desalting on a PD10 column. The apo-Csp3s were quantified using both the Bradford and DTNB assays, while the concentrations of Cu(I)-Csp3 samples were determined from the Bradford assay value corrected using the Bradford:DTNB ratio for the apo-sample. Far-UV CD spectra of the Csp3s (apo: 0.66–0.86 mg/mL, 46–67 μM; Cu(I): 0.68–0.84 mg/mL, 47–65 μM) were recorded aerobically on a Jasco J-810 spectrometer at 20 °C using a 0.2-mm-path-length quartz cuvette, as described previously.^13,40,53^

Analytical gel-filtration chromatography was performed on a Superdex 75 10/300 GL column (GE Healthcare) equilibrated in degassed 20 mM HEPES pH 7.5 plus 200 mM NaCl purged with nitrogen throughout the experiment.^13,51^ Protein concentrations ranged from 30 to 152 μM for apo-Csp3s and from 29 to 101 μM for the Cu(I) forms [binding ∼16–19 mol equiv of Cu(I)], in which the sample volume was 100 μL and the flow rate was 0.8 mL/min with elution monitored at 240 nm. Calibration of the column and the determination of estimated molecular weights were carried out as described previously.^13,51^

### Average Cu(I) Affinities

The average Cu(I) affinities of *Rk*Csp3 and *Sl*Csp3 were determined using an approach described previously.^13,31,37^*Rk*Csp3 and *Sl*Csp3 (∼2.5 μM) were incubated with increasing concentrations of Cu(I) in the presence of either 120–181 or 120 μM BCS, respectively. The absorbance of samples at 483 nm was measured at regular intervals for up to 120 h. The fractional occupancy of sites was plotted against the free Cu(I) concentration ([Cu(I)_free_]) and fit to the nonlinear form of the Hill equation providing *K*_Cu_ [the average dissociation constant for Cu(I)] and the Hill coefficient. To check the calculated maximum occupancies, appropriate samples were separated from [Cu(BCS)_2_]^3–^ and free BCS using a PD10 column in 20 mM HEPES pH 7.5 plus 200 mM NaCl in the anaerobic chamber. The resulting protein samples were analyzed for Cu(I) with BCS (∼2.5 mM) in the presence of 6.8 M guanidine hydrochloride and for total Cu by AAS. The protein concentration was determined with a Bradford assay corrected using the Bradford:DTNB ratio for the apo-protein sample used in the experiment.

### Cu(I) Removal from Csp3s by BCS

The rate of Cu(I) removal from almost fully Cu(I)-loaded Csp3s was investigated using BCS as described previously.^13,14^ Csp3s (1.1–1.3 μM) binding 15.9–17.1 (*Rk*Csp3) and 15.8–18.1 (*Sl*Csp3) mol equiv of Cu(I), prepared as described above, were mixed anaerobically with 2.5 mM BCS in 20 mM HEPES pH 7.5 plus 200 mM NaCl. The absorbance of the sample at 483 nm was monitored over time at 22 °C. Plots of [Cu(BCS)_2_]^3–^ concentration against time were fit to either one or two exponentials in Origin 7. The removal of Cu(I) was also performed in the presence of 6.8 M guanidine hydrochloride to determine the amount of Cu(I) present in the sample and therefore the expected end point for each reaction.

### Crystallization, Data Collection, Structure Solution, and Refinement for Cu(I)-*Rk*Csp3

For crystallization, apo-*Rk*Csp3 (69.5 μM) was incubated with ∼18 equiv of Cu(I) in 20 mM HEPES pH 7.5 plus 200 mM NaCl in the anaerobic chamber. The sample was concentrated using a Vivaspin 500 centrifugal concentrator (10 kDa MWCO). The final sample was quantified with a Bradford assay (17.8 mg/mL, obtained by correction using the Bradford:DTNB ratio), and the Cu(I) concentration was assayed using BCS in the presence of guanidine hydrochloride, giving 22.8 equiv. The Cu(I)-protein was removed from the anaerobic chamber, and screens were set up on a mosquito liquid-handling robot (SPT Labtech). Crystallization plates were immediately returned to the anaerobic chamber, sealed after being flushed with nitrogen for 3 min, and incubated at room temperature. Diffraction-quality crystals were obtained using the sitting drop method of vapor diffusion from 200 mM MgCl_2_, 100 mM HEPES pH 7.5 plus 25% PEG 3350 (100 or 200 nL of protein plus 100 nL of reservoir solution, well volume 80 μL). Crystals were cryoprotected with 25% ethylene glycol, and diffraction data were collected at the Diamond Light Source (Didcot, U.K.). Data were integrated with XIA2,^54^ using XDS,^55^ and scaled with Aimless.^56^ The space group was confirmed using Pointless.^57^ Phases were solved by molecular replacement using Phaser^58^ and PDB file 3LMF as the search model. The model was refined with refmac^59^ and manual model building was carried out with COOT.^60^ Model validation was performed with COOT^60^ and Molprobity.^61^ Other software used was from the CCP4 cloud and the CCP4 suite.^62,63^ Figures were made with PyMol,^64^ and data collection statistics and refinement details are reported in Table S3.

## Data Availability

The raw data underpinning conclusions of this article that are not included in the Supporting Information will be made available by the authors.

## Supporting Information Available

Supporting results, amino acid sequence alignments of Csp3s, Cu(I) binding data for *Rk*Csp3 and *Sl*Csp3, average Cu(I) affinity for *Sl*Csp3, tetrameric arrangement of crystallized Cu(I)-*Rk*Csp3, influence of Cu(I) on secondary and quaternary structures of *Rk*Csp3 and *Sl*Csp3, an overlay of the structures of Cu(I)-*Rk*Csp3 and apo-*Nm*Csp3, fits of Cu(I) uptake and removal data, and data statistics and refinement for the crystal structure of Cu(I)-*Rk*Csp3 (PDF)

## Accession Codes

The crystal structure of Cu(I)-*Rk*Csp3 has been deposited at the Protein Data Bank with the code 6ZIF.
