# Supplementary material for: Important Structural Features of Thiolate-Rich Four-Helix Bundles for Cu(I) Uptake and Removal
Source: Inorg Chem. 2023 Apr 14;62(17):6617–28. doi: 10.1021/acs.inorgchem.2c04490 (PMC10155185; doi:10.1021/acs.inorgchem.2c04490)
Supplement: Supplementary file 1 — ic2c04490_si_001.pdf [file ic2c04490_si_001.pdf]

SUPPORTING INFORMATION

# Important Structural Features of Thiolate-Rich Four-Helix Bundles for Cu(I) Binding and Removal

*Jaeick Lee, Rosemary A. Dalton, Arnaud Baslé, Nicolas Vita, and Christopher Dennison**

Biosciences Institute, Newcastle University, Newcastle upon Tyne NE2 4HH, UK. E-mail: christopher.dennison@ncl.ac.uk

## SUPPORTING RESULTS

**The Binding of Cu(I) to *Rk*Csp3 and *Sl*Csp3**. The binding of Cu(I) to *Rk*Csp3 was analysed by monitoring the appearance of S(Cys)→Cu(I) ligand-to-metal charge transfer (LMCT) bands below 400 nm (Figure S2A). Plots of absorbance at selected wavelengths against the amount of the Cu(I) added (Figure S2B) provide approximate stoichiometries of ~18.5 to 20.0 (n = 2). Emission at around 600 nm (Figure S2C), another characteristic feature of Cu(I) thiolate clusters found within the cores of Csp3s,[1] reaches a maximum after 12.5 Cu(I) equivalents are added to the protein (Figure S2D). This is slightly higher than for *Mt*Csp3,[1] and *Sl*Csp3 (see below), which reach a maximum emission at ~10 Cu(I) equivalents. A more precise measure of the number of Cu(I) equivalents bound by *Rk*Csp3 is obtained by adding the metal ion in the presence of approximately a 40-fold excess of bicinchoninic acid (BCA) (Figure 1A). This gives 18.0 ± (standard deviation) 0.6 (values ranging from 17.5 to 18.9, n = 5) for mixtures incubated for 48 h.

The formation of S(Cys)→Cu(I) LMCT bands upon titrating Cu(I) into *Sl*Csp3 (Figure S3A) looks similar to previously reported data.[2] Our results give Cu(I)-binding stoichiometries of ~20-21 (n = 2) at selected wavelengths (Figure S3B), like those for *Mt*Csp3,[1] and also *Rk*Csp3. The emission at around 600 nm reaches a maximum after ~8-10 equivalents of Cu(I) were added to *Sl*Csp3 (Figure S3C,D). This is similar to *Mt*Csp3,[1] but the emission values are significantly lower than for *Mt*Csp3 and *Rk*Csp3, and are like those for *Bs*Csp3.[1] The more precise measure of Cu(I) ions bound by *Sl*Csp3 (obtained by adding Cu(I) in the presence of approximately a 40-fold excess of, Figure S4), gave 18.2 ± 0.5 (n = 3) equivalents. This is comparable to data for *Mt*Csp3 (17.9 ± 1.0),[1] consistent with crystal structures of the Cu(I)-proteins.[1,2]

**The Average Cu(I) Affinities of *Rk*Csp3 and *Sl*Csp3**. The average association constant of *Rk*Csp3 is $(8.2 \pm 1.1) \times 10^{17}$ $M^{-1}$ [a $K_{Cu}$ of $(1.2 \pm 0.2) \times 10^{-18}$ M], with a Hill coefficient of 1.7 ± 0.5 (n = 3) (Figure 1B,C). For *Sl*Csp3, an average Cu(I) affinity of $(1.6 \pm 0.1) \times 10^{17}$ $M^{-1}$ [$K_{Cu}$ of $(6.0 \pm 0.4) \times 10^{-18}$ M] and a Hill coefficient of 1.0 ± 0.1 (n = 3) were determined (Figure S5). These data are very similar to those for *Mt*Csp3 [$(1.7 \pm 0.5) \times 10^{17}$ $M^{-1}$ and a Hill coefficient of 1.0 ± 0.1].

**Cu(I) removal from *Sl*Csp3**. In the presence of 2.5 mM bathocuproine disulfonic acid (BCS) 57.3-68.0 % (n = 3) of Cu(I) was removed from *Sl*Csp3 after 85 h (Figure 5B). The speed of Cu(I) removal by this high affinity ligand is much slower than for *Rk*Csp3 (Figure 5A, Table S2). However, Cu(I) removal is faster than from *Mt*Csp3,[1] and is similar to that measured for *Bs*Csp3 (Table S2).[1]

**The Influence of Cu(I)-Binding on the Structure of *Rk*Csp3 and *Sl*Csp3**. The α-helical contents of apo- and Cu(I)-*Rk*Csp3, measured by far-UV circular dichroism (CD) spectroscopy (Figure S7A), are 67.6 ± 2.5 and 66.1 ± 1.1 %, respectively (n = 3), consistent with the crystal structure (~70% α-helix). The α-helical contents of apo- and Cu(I)-*Sl*Csp3 measured by far-UV CD (Figure S8A) are 85.9 ± 1.3 % and 83.4 ± 1.2 %, respectively (n = 3). This is similar to the amount of α-helix calculated from crystal structures[2] (PDBs: 6EI0 and 6EK9, ~80%).

Apo-and Cu(I)-*Rk*Csp3 eluted as tetramers from an analytical gel-filtration column (Figure S7B), with apparent molecular weights of 45 ± 1 and 44 ± 1 kDa, respectively (both n = 3). These values are slightly smaller than the calculated molecular weight of an *Rk*Csp3 tetramer (51.5 kDa). Apo-*Sl*Csp3 eluted from an analytical gel-filtration column largely as a single peak (Figure S8B) with an apparent molecular weight of 55 ± 1 kDa (n = 3), very similar to the calculated value for a *Sl*Csp3 tetramer (57.3 kDa). The shoulder at a larger elution volume (~11-11.5 mL) for apo-*Sl*Csp3 indicates dissociation of the tetramer as seen previously for apo-*Mt*Csp3.[1] Cu(I)-*Sl*Csp3 elutes as clear single peak (Figure S8B), but with a smaller apparent molecular weight of 50 ± 1 kDa, indicating the *Sl*Csp3 tetramer may become more compact upon Cu(I) binding.

## Multiple sequence alignment of classical Csp3s from a range of bacteria

```
                                                   24               41      49   54  58
Methylosinus      M--H--VEAMISKHPQ--ARG--Q-T-DRSLVQCVEMCFDCAQTCAACADACLGEDKVADLRHCIRLNLDCAEICVAAGS 70
Chthonobacter     M--H--VQSMISTHPH--VKG--N-T-NDALIRCIEECYDCAQTCTSCADACLGEDMVKDLTQCIRLNLDCADVCAATGA 70
Pleomorphomonas   M--H--TEAMIRSHPA--LNG--T-V-DNDLLHCIEACLDCAQTCTSCADACLGEQNVDDLRQCIRLNLDCADLCLATAT 70
Methylocaldum     M--NA-IQEMLKAHPHPAVMGRGV-D-MGLLSQTASELFDCAQACTACADACVGEDQVQNLKRCIRMCMDCADICVATGR 75
Streptomyces      M--PTTVNDLLRTYPA--DLG--GVD-REAMARCIEECLRCAQACTACADACLSEPTVADLTKCIRTDMDCADVCTATAA 73
Bacillus          M--E--Q---------------------YSEACIEACIDCMKACNHCFTKCLEESVQHHLSGCIRLDRECADICALA-- 52
Azospirillum      M--H--ARAMISTHPQ--VRG--N-T-NDALIRCIESCYDCAQTCTTCADACLGEDQVAELVQCIRLNMDCADVCTATGS 70
Phenylobacterium  M--H--AHEIISTHPQ--VRG--N-T-NDSLIRCIEECFDCAQSCTSCADACLGEEMVAQLTQCIRLNLDCADICTAAGH 70
Caulobacter       M--H--AHEIISTHPQ--VRG--A-T-NDSLIRCIEECFDCAQSCTSCADACLGEQMVAQLTQCIRLNLDCADICTAAGH 70
Microvirga 1      M--H--VQEMISTHPH--VKG--D-T-NNALIRCIEECYSCAQVCTSCADACLGEDMVQRLTQCIRLDLDCADVCAATGS 70
Rhizobium 1       M--H--VREMISTHPH--VRG--E-T-EDALLRCIEECYACAQTCTSCADACLGEENTGMLIQCIRRNLDCADLCAATGA 70
Bradyrhizobium 1  M--H--AQEMISTHPQ--VRG--Q-T-NDALIRCIEECYSCAQTCTSCADACLAENNVKSLTQCIRLNLDCADICNITGR 70
Skermanella       M--L--VREMIGTHPD--VKG--S-I-NDALIRCIEECYSCAQTCTSCADACLAEDMVKELRQCIRLNLDCADICAAAGS 70
Paracoccus 1      M--H--IQQMIATHPE--VKG--H-T-AGRLLRCIEECHDCAAVCTSCADACLAEPMVEQLRQCIRLNLDCADICATAGA 70
Arthrobacter      MTHH--VTSMLDTYPK--DLG--GVD-KEKLAQCIQACFECAQTCTACADACLSEDMVADLTKCIRTNLDCADICVTTGN 73
Sphingobium       M------QEMIATHPE--VKG--N-T-NDALIRCIEECYSCAQICTSCADACLAEEMVAQLRQCIRLNLDCADICLAAGS 68
Janibacter        M--HT-VEQMLDTYPK--DLG--GID-RAKLTECIQACFECAQTCTACADACLSEDKVADLTQCIRANLDCADICTTTGS 72
Corynebacterium   MPHH--VHAMLETHPK--DLG--Q-IDKDKLAECITACFECAQTCTACADACLGEDMVAELTTCIRLNLDCADICTATGR 73
Rubrivirga        M--R--TQDMLSKHSD--PSD--Q--L-DKVVALVNSAFACEQCCTSCADACLAEDSDMDLTACIRTNLDCADVCATTGR 69
Cellulomonas      ---------MLDTYPA--TIN---LD-RQLLARVIESLVACSQACTACADACLSEEMVADLRKCIRSNLDCADSCAATAR 65
consensus                                                 *   *  *   *: *     *  ***   :**: *  :
helices             ----αN----              ---------α1---------      -------α2-------
```

```
                       76         87               104 108 110
Methylosinus      IASRAAGTEESILRTMLQTCAEMCRMCEEECRRHAGNHEHCRICADVCKECETACRSATGLTH----- 133
Chthonobacter     VASRRTGSNESVIKMMIEACAEACRLCGEECARHAQMHEHCRICAEACRRCEEACRQASGTITPRMQ- 137
Pleomorphomonas   IASRRTGSNGEVLRAVILACRDACRACAAECGRHATMHEHCRVCADACRHCEDACATAADGIKSSLQ- 137
Methylocaldum     LVSRETESDMRMMRSQLQACAAACGICAEECERHASRHEHCRICGESCRRCEQACNKLAGAITM---- 139
Streptomyces      VLSRHTGYDANVTRAVLQACATVCAACGDECARHAGMHEHCRVCAEACRSCEQACQELLAGLG----- 136
Bacillus          --VKAMQTDSPFMKEICALCADICEACGTECGKHD--HDHCQACAKACFTCAEQCRSMAA-------- 108
Azospirillum      VATRRSGSNEAVIRAMLDACATACRLCAEECERHAGMHQHCRICAEACRTCEDACRKALQTLS---H- 134
Phenylobacterium  IASRRTGSNEPIIQSVLRACEEACRLCAEECERHASMHEHCRICAESCRRCEQACQAALQTF----H- 133
Caulobacter       IASRRTGSNEPIIQSVLRACEEACRLCAEECDRHASMHEHCRICAESCRRCEQACQAALQTF----H- 133
Microvirga 1      VATRRTGSNEEVIRQMLEACATACRLCAEECESHAGMHEHCRICAESCRRCEQACQSALGSMGR---- 134
Rhizobium 1       IASRRTGSNTEVMRAVIQACAEACRVCGEECRSHAEMHEHCRVCADACERCEKACREALPAVH----- 133
Bradyrhizobium 1  ISTRRTGSDEEMIRRMLDTCAAACRLCGEECEKHAKMHEHCRICAESCRRCMNACEDAGRSMV---H- 134
Skermanella       IATRRTGSNEELIRLTLQACATACRLCGAECDSHASMHEHCRICAESCRRCEQACQAALQSMAA---- 134
Paracoccus 1      IASRRTGSNIEVIRAVIDACGTACRACGEECRKHAGMHEHCRICAESCMRCAEACRDALGDLH----- 133
Arthrobacter      ILSRHTGYDANLTRAVLEACRTACKACGDECERHAGMHEHCRVCAEACRRCETACQELLSSLG----- 136
Sphingobium       LGTRRTGSNEQALVAALQACAIACGLCAEECEKHASTHEHCRICAEHCHRCEQACSEAVQSIR----- 131
Janibacter        ALSRHTGYDANVTRALLEACATACTSCGDSCEQHASMHEHCRICAEACRRCERACRELISSLT----- 135
Corynebacterium   MLSRPTDWNVTLIRSVLEACRTACQACGEECARHAEMHEHCTVCAEACRRCEQACTELLATL-----D 136
Rubrivirga        VLSRQTQPNDAILRAQLQACVAACDACADECEEHADMHEHCRVCMECCRECADACRALLDAMPQGATA 137
Cellulomonas      ILSRHTGYDANITRAHLEACIAACRACGDECEQHAGMHEHCRICAEACRDCETACAELLAAIR----- 128
consensus            :    :          *   *  *  .*  *   *:**  * . *  *   *
helices           ---         ------α3------  ------α4------
```

## Multiple sequence alignment of the new (*Rk*Csp3) sub-family of Csp3s from a range of bacteria

```
                                       11                29      37   42  46            60
Methylocystis     MHK-----------MSKEMQSCVDECLRCYQMCFGMAMTHCLETGGDHVKPKHFRAMISCAEMCRNAAHMMLMKSPQARH 69
Methylotetra      MNHPD--------QTEDAMQECIDACSQCHQVCLETAMNHCLETGGKHVKPKHFRLLMNCAEICQLSANFQLSNSHFQHR 72
Methylomicro      MYTPQTEPQKTSTAAFQSMQPCIDNCNRCAQTCLQTAMNQCLEMGGRHVEPEHFRLMICCAEICRLSANFMLSSSPFHTR 80
Methylobacter     -----------------MQSCIEMCTRCSQVCRQTAMNQCLEMGGQHVEPEHFRLMMNCAEICQTSANFMLSSSRFHNR 62
Methylomonas      MSQSS--------HSEHATQTCIDACNLCHQICLHTAMTHCIETSGKHVKAKHLRLMINCAEICQTTGNFQLTGSHFLPR 72
Methylococcus     MHQPA--------LTEHAMQACIEACSHCHQICLHTAMNHCLKAGGKHVKAKHFRLLMNCAEICQLSANFQLGSSVFQHR 72
Nitrosospira      MFLYT--------ETDQNLQACIDACNHCYRTCLRMAMNHCLEAGGKHVEADHLRLMMNCAEICQTSLNFMLSGSRFSPK 72
Afipia            MNQ-----------MSKEMQSCIDECLRCYQVCLGTAMTHCLETGGKHVEPQHFRLMMACAEICRTAAHFMLLNTPHHKH 69
Bradyrhizobium 2  MK------------TSKEMQACIDECLRCYQMCLGTAMTHCLEMGGEHVEPPHFRLMMACSEMCRTAAHFMLLNSPHHAH 68
Paracoccus 2      MHA-----------MTPEMQSCIDECLACYRTCLGEAMNHCLEAGGKHTEPVHFRTMIACAEMCRTSAHLMLTGSELHRH 69
Rhizobiales       MKH-----------ISPEMKTCIDHCLACYRECLSTAMGHCLETGGRHTEPSHFRLMSACAEICRTAAHFMLIGSQHHRH 69
Microvirga 2      MHQ-----------MSKEMQSCIDECLRCYQTCLGMAMTHCLEQGGKHVEPGHFRLMMACAEICRTSAHFMLIGTPHHRH 69
Nordella          MQH-----------ISREMQSCIKECLNCYQACVSTAMTHCLEKGGEHTKPEHFRLISACAEICRTAAHFMLINSPQHKG 69
Pigmentiphaga     MNA-----------VPKEMQLCVDACLRCYQSCLGSAMNHCLERGGAHVAPPHFRLMMACAEMCRTAAHFMLTGVPQHRH 69
Roseomonas        MHN-----------MSPEMQACIEECLRCHSTCLGMAMNHCLEVGGKHVEPEHFKLMMACAEICQTSANMMLIGTHHHKH 69
Methylobacterium  MHQ-----------TSPEMQACIDECLRCYQACLGMSTNHCLKLGGDHASPDHIQLMLACAETCRASAQVMIIGSRHHKH 69
Siccirubricoccus  MQK-----------LSPELRSCIEECLRCHGTCLGMAMNHCLEQGGKHLEPRHFRLMLACAEICQTAANFMLIGTPHHKH 69
Inquilinus        MHQ-----------LSTDIRSCIDDCTRCYQTCLSTAMLHCLEAGGQHVEPAHFRLMAACAEICRAAAHVMLTGTEHHRR 69
Rhizobium 2       MHH-----------MSTEMKACIDNCLACYGECLSMAMGHCLELGGEHTKPAHFKLMMACAEICRTSAHFMLIGSEHHKH 69
Fischerella       MAIQQLT----LNQVNQGMQECIQNCLDCHSICLNT-VTYCLQKGGNHAEQSHIRLLLDCAEICETSANFMLRTSQLHAR 75
consensus                            : *:. *  *   *       *:: .* *    *:: :  *:* *. : :. :
helices                          ---------α1---------         -------α2-------  ---
```

```
                      71                  88 90    91                108
Methylocystis        ICEDCAEACEACAKECDALPD---MKDCAAQCRRCAEACRKMAGQKMAA 115
Methylotetra         LCEVCAEVCEACADDCEKLGD---MEECIEACRECAESCRKMASMK--H 116
Methylomicro         TCEVCAEICEACAKDCASIGD---MDECVSICRECAESCKQMASMAT-H 125
Methylobacter        TCEVCAEICEACAMDCDSIGD---MEECASTCRECADSCRQMAGMTTQH 108
Methylomonas         LYELCAEICEACAAECDEIGD---MNDCAVACQKCADRCRQMLGAQ--Q 116
Methylococcus        LCAICAEVCEACATDCEEIGD---MDECVQACRECAESCRQMAAVE--H 116
Nitrosospira         VCGVCAEICDACAKSCEQLDG---MEECVQTCRQCAEHCRKMAA----- 113
Afipia               VCGECAEICTECAKDCERIGS---MDDCVKACKQCAESCRAMA-----G 110
Bradyrhizobium 2     LCEECAEICTECAKDCERVGG---MDDCVTACTRCAESCRAMA-----G 109
Paracoccus 2         SCAACAEICEACAESCEGIEG---MEACAEQCRRCAESCRHMA-----A 110
Rhizobiales          VCRECAEICEECARDCEAIGD---MQSCVDACRRCAESCRKMAA----- 110
Microvirga 2         TCRECAEICEECAKSCEQVGD---MQECVDQCRRCAESCRKMAA----- 110
Nordella             ICAECADICKQCAEDCERIGD---MADCVEACRRCTESCRTMAAGHRAA 115
Pigmentiphaga        VCADCASICRQCADSCDGLDG---MDRCAADCRSCADACERMAA----- 110
Roseomonas           TCRECAEICEECAKSCDAVGG---MEECAEQCRKCAASCRAMAA----- 110
Methylobacterium     LCAECADICEDCANECERIGD---MDDCVRACRRCAESCRKMAA----- 110
Siccirubricoccus     TCGECAEICEECASSCEQVGE---MEECVQACRSCAESCRKMAA----- 110
Inquilinus           VCGACAEICDACATDCERIGD---MQDCVDACRRCAASCRKMAA----- 110
Rhizobium 2          VCPECAEICAQCADDCERIGD---MQGCVDACRRCAESCRKMAA----- 110
Fischerella          TCGVCAEVCERCAENCDRMGDDAQMKACADMCRRCAESCRQMSMAT--A 122
consensus                **. *  ** .*  :     *  *   *  *:  *. *
helices              ------ α3 -------   -------- α4 --------
```

## Amino acid sequence alignment of *Mt*Csp3, *Sl*Csp3, *Bs*Csp3 and *Rk*Csp3

```
                                                24               41      49   54   58                 76
Methylosinus         MHVE--AMISKHPQARGQTDR-SLVQCVEMCFDCAQTCAACA-DACLGEDKVADLRHCIRLNLDCAEICVAAGSIASRAA 76
Streptomyces         MPTTVNDLLRTYPADLGGVDREAMARCIEECLRCAQACTACA-DACLSEPTVADLTKCIRTDMDCADVCTATAAVLSRHT 79
Bacillus             ME-----------------Q-YSEACIEACIDCMKACNHCF-TKCLEESVQHHLSGCIRLDRECADICALAVKAM--- 56
Methylocystis        MHK---------------MSK-EMQSCVDECLRCYQMCFGMAMTHCLETGGDHVKPKHFRAMISCAEMCRNAAHMM--- 60
consensus            *                   :     *::  *: * : *       **           :*   .**::*  :
helices 1              --- αN ---            -------- α1 --------             ------ α2 ------
helices 2                                     ------- α1 -------               ------ α2 -------
```

```
                               87                   104  108 110
Methylosinus         GTEESILRTMLQTCAEMCRMCEEECRRHAGNHEHCRICADVCKECETACRSATG-LT--H 133
Streptomyces         GYDANVTRAVLQACATVCAACGDECARHAGMHEHCRVCAEACRSCEQACQELLAGL---G 136
Bacillus             QTDSPFMKEICALCADICEACGTECGKHD--HDHCQACAKACFTCAEQCRSMA------A 108
Methylocystis        LMKSPQARHICEDCAEACEACAKECDALP----DMKDCAAQCRRCAEACRKMAG-QKMAA 115
consensus              .    : :   **  *  *  **        . : **  *  *   *:.
helices 1                ------ α3 -------          ------- α4 -------
helices 2            --  ----- α3 -----              ------ α4 ------
```

**Figure S1.** Multiple sequence alignments of Csp3s produced using T-coffee.[3] The first alignment compares a number of classical Csp3s from a range of bacteria. This is followed by an alignment of Csp3s that belong to the *Rk*Csp3 sub-class also from a range of bacteria. The final alignment compares the fours Csp3s (*Mt*Csp3, *Sl*Csp3, *Bs*Csp3 and *Rk*Csp3) that are studied in this work. Asterisks denote fully conserved sequence positions; the ':' and '.' symbols indicate strongly and weakly similar sequence positions, respectively. Cysteine residues are highlighted yellow, whilst other key residues are highlighted cyan. The names of methanotrophs are orange and those of other bacteria are black. The α-helices based on the structure of *Mt*Csp3 [2] are used in all three sequence alignments (helices 1 in the final alignment), whilst those present in the structure of Cu(I)-*Rk*Csp3 are shown in the final alignment as helices 2. The names of the bacterial sources of the sequences

are as found on the NCBI website using the listed protein accession code: Methylosinus - *Methylosinus trichosphorium* OB3b, accession WP_003608458.1; Chthonobacter - *Chthonobacter rhizosphaerae* yh7-1, accession WP_181706096.1; Pleomorphomonas - *Pleomorphomonas koreensis* DSM 23070, accession WP_210162665.1; Methylocaldum - *Methylocaldum marinum* S8, accession WP_119628450.1; Streptomyces - *Streptomyces lividans* 1326, accession WP_003975554.1; Bacillus - *Bacillus subtilis* 168, accession WP_009966950.1; Azospirillum - *Azospirillum brasilense* Az19, accession WP_149650126.1; Phenylobacterium - *Phenylobacterium sp.* SCN 70-31, accession ODT84700.1; Caulobacter - *Caulobacter sp.* CCH9-E1, accession WP_066680677.1; Microvirga 1 - *Microvirga guangxiensis* CGMCC 1.7666, accession WP_091135858.1; Rhizobium 1 - *Rhizobium pseudoryzae* DSM 19479, accession WP_165217320.1; Bradyrhizobium 1 - *Bradyrhizobium sp.* WSM1253, accession WP_007602726.1; Skermanella - *Skermanella stibiiresistens* SB22, accession WP_084164105.1; Paracoccus 1 - *Paracoccus salipaludis* WN007, accession PAU96203.1; Arthrobacter - *Arthrobacter sp.* B3, accession WP_104443547.1; Sphingobium - *Sphingobium sp.* ba1, accession WP_202807683.1; Janibacter - *Janibacter sp.* YB324, accession WP_084453131.1; Corynebacterium - *Corynebacterium sp.* L2-79-05, accession WP_173051580.1; Rubrivirga - *Rubrivirga marina* SAORIC-28, accession WP_095510004.1; Cellulomonas - *Cellulomonas flavigena* DSM 20109, accession WP_043599515.1. Methylocystis - *Methylocystis sp.* ATCC49242 (Rockwell), accession WP_036288389.1; Methylotetra - *Methylotetracoccus oryzae* C50C1, accession WP_139559480.1; Methylomicro - *Methylomicrobium album* BG8, accession EIC28528.1; Methylobacter - *Methylobacter whittenburyi* ACM3310, accession WP_036294836.1; Methylomonas - *Methylomonas koyamae* LM6, accession WP_096875976.1; Methylococcus - *Methylococcus sp.* EFPC2, accession WP_205433248.1; Nitrosospira - *Nitrosospira multiformis* ATCC 25196, accession WP_011381062.1; Afipia - *Afipia clevelandensis* ATCC49720, accession WP_002714854.1; Bradyrhizobium 2 - *Bradyrhizobium sp.* UASWS1016, accession WP_100554758.1; Paracoccus 2 - *Paracoccus salipaludis* WN007, accession

WP_095641146.1; Rhizobiales - *Rhizobiales bacterium*, accession WP_112827010.1; Microvirga 2 - *Microvirga tunisiensis* M8, accession WP_152710736.1; Nordella - *Nordella sp*. HKS 07, accession WP_165166511.1; Pigmentiphaga - *Pigmentiphaga kullae* K24, accession WP_130360151.1; Roseomonas - *Roseomonas mucosa* AU37, accession WP_058389173.1; Methylobacterium - *Methylobacterium sp.* CCH7-A2, accession WP_054207532.1; Siccirubricoccus - *Siccirubricoccus phaeus* 1-3, accession WP_149538236.1; Inquilinus - *Inquilinus limosus* DSM 16000, accession WP_026871166.1; Rhizobium 2 - *Rhizobium azibense* Gr42, accession WP_132661307.1; Fischerella - *Fischerella thermalis* CCMEE 5205, accession WP_102184949.1.

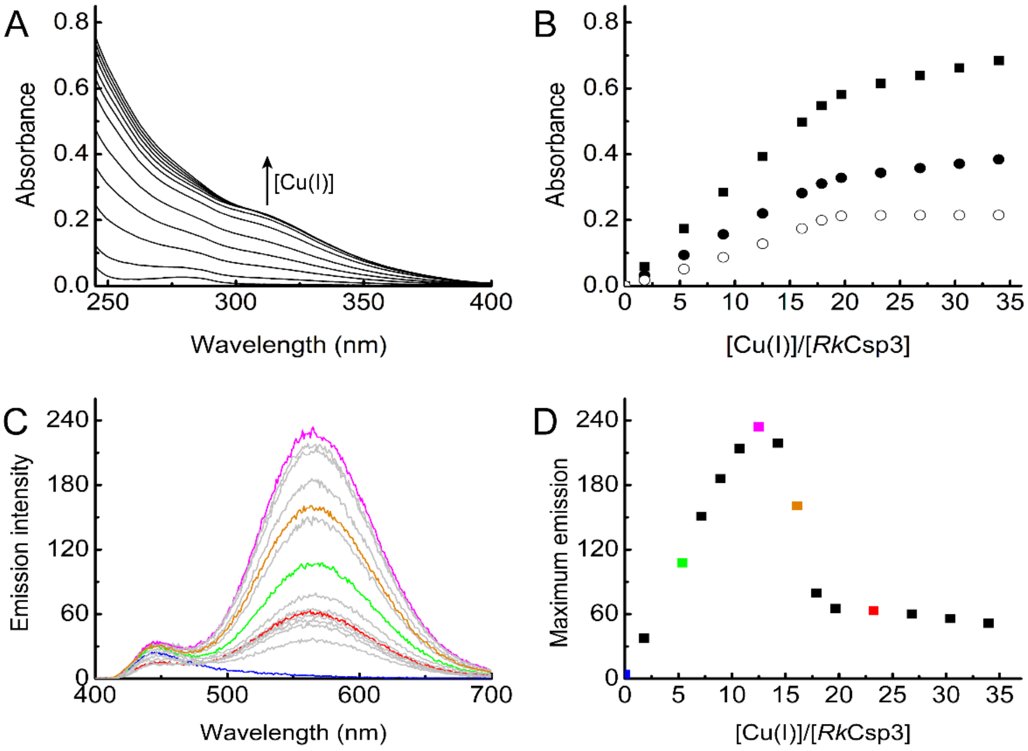


**Figure S2.** Cu(I) binding to *Rk*Csp3 monitored by UV/vis and fluorescence spectroscopy. (A) UV/vis difference spectra obtained upon the addition of Cu(I) to apo-*Rk*Csp3 (5.00 µM), and (B) plots of the absorbance at 250 (filled squares), 275 (filled circles), and 315 (open circles) nm against the [Cu(I)]/[*Rk*Csp3] ratio (for a monomer) from the spectra shown in (A). (C) Emission spectra after excitation at 280 nm upon the titration of Cu(I) into apo-*Rk*Csp3 (5.00 µM). (D) A plot of the maximum emission (between 550 and 600 nm) against the [Cu(I)]/[*Rk*Csp3] ratio (for a monomer) from the spectra shown in (C). In (C) and (D) the data at 0 (blue), 5.4 (green), 12.5 (magenta), 16.1 (orange), and 23.2 (red) equivalents of Cu(I) are highlighted. Experiments were performed twice in 20 mM HEPES pH 7.5 plus 200 mM NaCl.

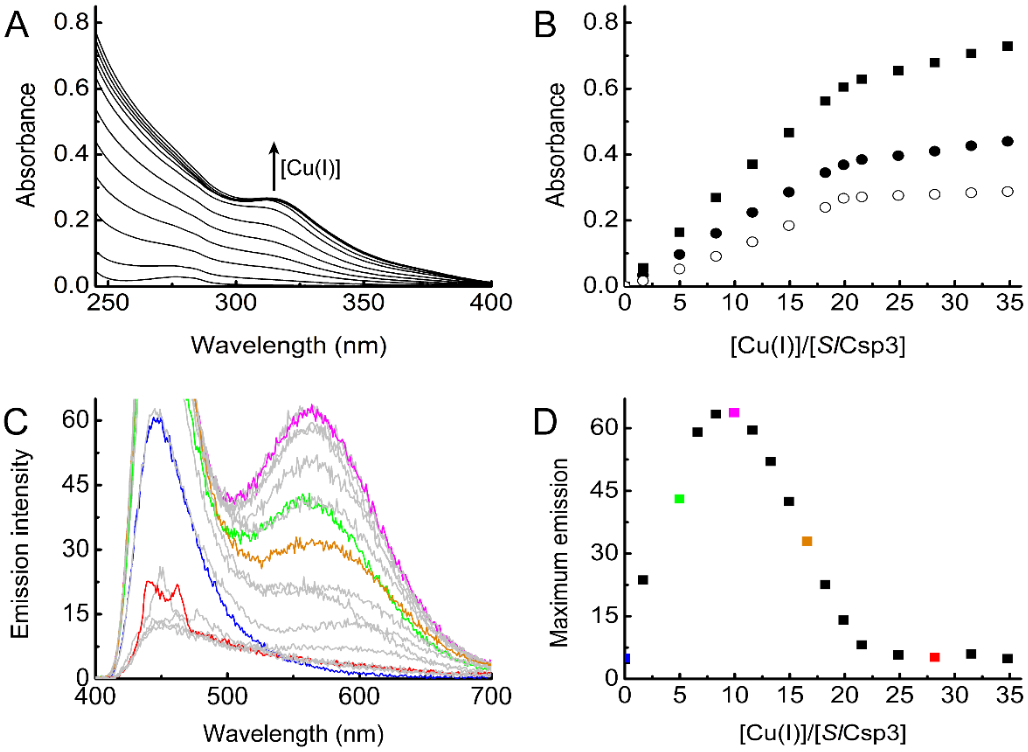


**Figure S3.** Cu(I) binding to *Sl*Csp3 monitored by UV/vis and fluorescence spectroscopy. (A) UV/vis difference spectra obtained upon the addition of Cu(I) to apo-*Sl*Csp3 (5.07 µM), and (B) plots of the absorbance at 250 (filled squares), 275 (filled circles), and 315 (open circles) nm against the [Cu(I)]/[*Sl*Csp3] ratio (for a monomer) from the spectra shown in (A). (C) Emission spectra after excitation at 280 nm upon titration of Cu(I) into apo-*Sl*Csp3 (5.07 µM). (D) A plot of the maximum emission (between 550 and 600 nm) against the [Cu(I)]/[*Sl*Csp3] ratio (for a monomer) from the spectra shown in (C). In (C) and (D) the data at 0 (blue), 5.0 (green), 9.9 (magenta), 16.6 (orange) and 28.2 (red) equivalents of Cu(I) are highlighted. Experiments were performed twice in 20 mM HEPES pH 7.5 plus 200 mM NaCl.

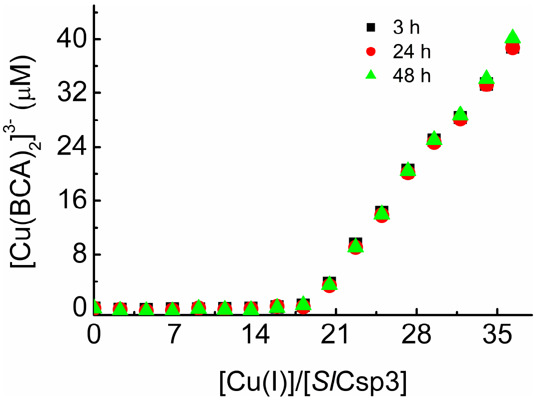


**Figure S4.** The Cu(I)-binding stoichiometry of *Sl*Csp3. A plot of $[Cu(BCA)_2]^{3-}$ concentration against the [Cu(I)]/[*Sl*Csp3] ratio (for a monomer) of mixtures of apo-*Sl*Csp3 (2.49 μM) and Cu(I) in the presence of 100.0 μM BCA incubated for 3, 24 and 48 h. The protein was in 20 mM HEPES pH 7.5 plus 200 mM NaCl and this experiment gave 18.4 equivalents of Cu(I) bound to the protein after equilibration for 48 h.

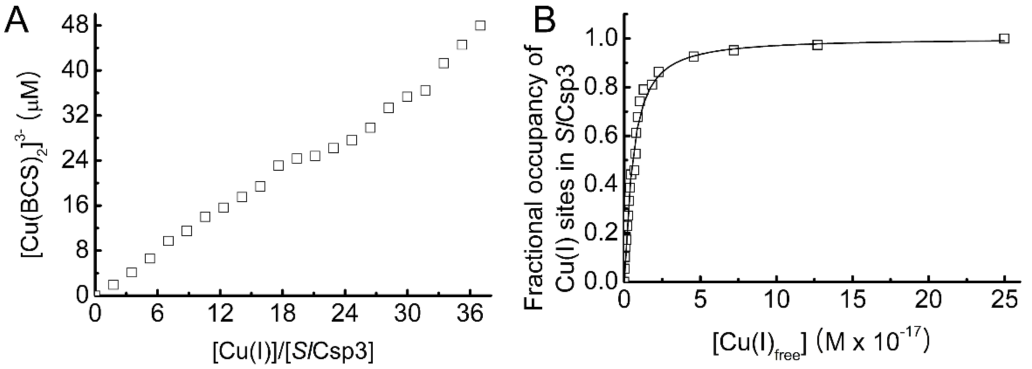


**Figure S5.** The estimated average Cu(I) affinity of *Sl*Csp3. (A) A plot of $[Cu(BCS)_2]^{3-}$ concentration against the [Cu(I)]/[*Sl*Csp3] ratio (for a monomer) for mixtures of apo-*Sl*Csp3 (2.54 μM) and Cu(I) in the presence of 120 μM BCS incubated for 96 h. (B) A plot of the fractional occupancy of Cu(I)-binding sites in *Sl*Csp3 at different $[Cu(I)]_{free}$ concentrations calculated from the data in (A). The maximum calculated value is 18.6 equivalents of Cu(I) per monomer, and experimental values of 18.6 (BCS) and 16.1 (atomic absorption spectroscopy) were determined using the final two samples. The solid line in (B) shows a fit of the data to the nonlinear Hill equation giving an average dissociation constant for Cu(I), $K_{Cu}$, of $(5.5 \pm 0.2) \times 10^{-18}$ M and Hill coefficient of $1.2 \pm 0.1$. The experiment was performed in 20 mM HEPES pH 7.5 plus 200 mM NaCl.

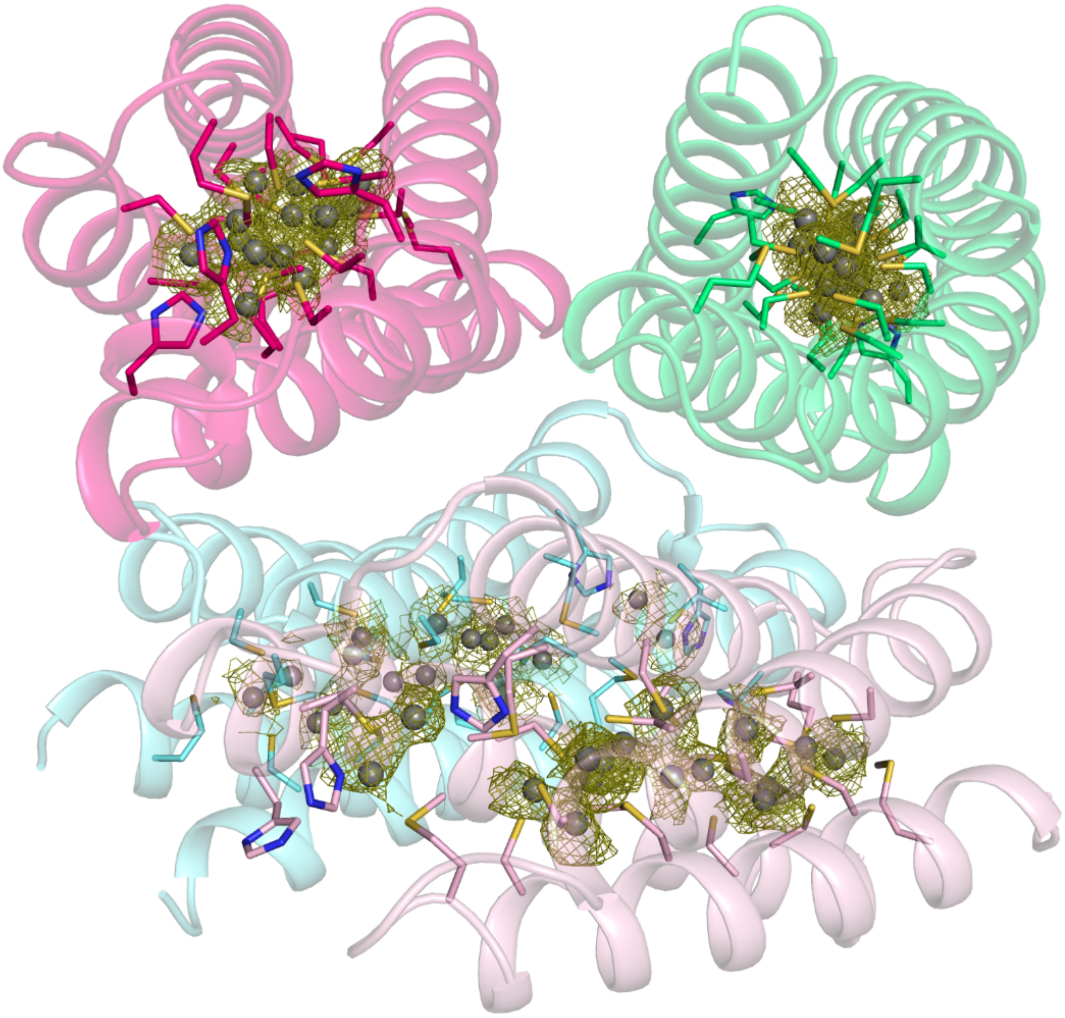

**Figure S6.** Copper anomalous difference density for the Cu(I)-*Rk*Csp3 tetramer. Each of the four-helix bundles that make up the Cu(I)-*Rk*Csp3 tetramer are shown in a different colour with the Cu(I) sites as grey spheres and the same side chains as in Figure 2A highlighted as sticks. Also shown is the anomalous difference density for copper contoured at 2.2 σ (olive mesh).

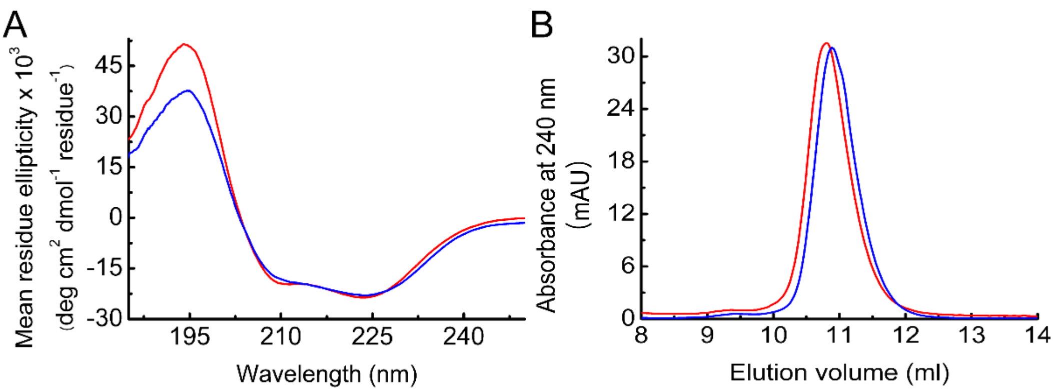


**Figure S7.** Secondary and quaternary structures of apo- and Cu(I)-*Rk*Csp3. Far-UV CD spectra of (A) apo-*Rk*Csp3 (61.6 µM, 0.79 mg/mL, red line) and protein (60.5 µM, 0.78 mg/mL) loaded with 16.6 equivalents of Cu(I) (blue line) per monomer. (B) Analytical gel-filtration chromatograms of apo-*Rk*Csp3 (101 µM, red line) and *Rk*Csp3 (95.1 µM) to which 16.0 equivalents of Cu(I) were added (blue line) per monomer. Far-UV CD samples were measured in 20 mM phosphate pH 8.0 and gel-filtration chromatography was performed in degassed and nitrogen bubbled 20 mM HEPES pH 7.5 plus 200 mM NaCl. The absorbance for Cu(I)-*Rk*Csp3 is divided by ~7 in (B).

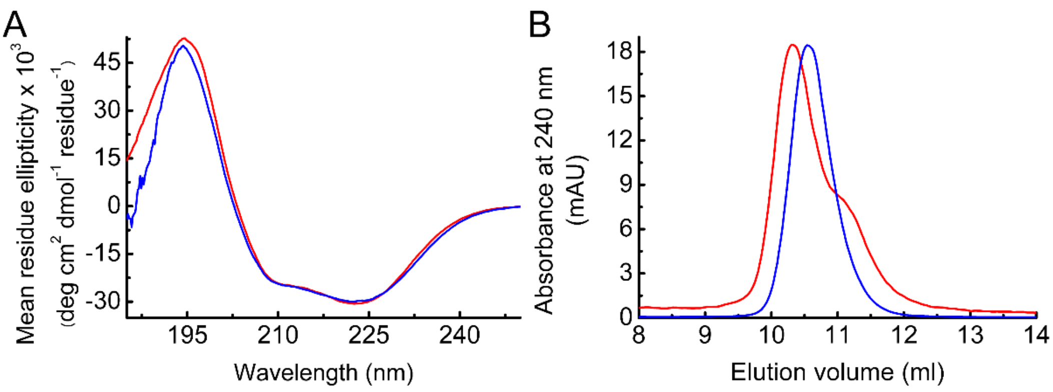


**Figure S8.** Secondary and quaternary structures of apo- and Cu(I)-*Sl*Csp3. Far-UV CD spectra of (A) apo-*Sl*Csp3 (48.2 μM, 0.69 mg/mL, red line) and of *Sl*Csp3 (49.6 μM, 0.71 mg/mL) loaded with 18.1 equivalents of Cu(I) (blue line) per monomer. (B) Analytical gel-filtration chromatograms of apo-*Sl*Csp3 (101 μM, red line) and *Sl*Csp3 (101 μM) to which 18.5 equivalents of Cu(I) were added (blue line) per monomer. The far-UV CD samples were measured in 100 mM phosphate pH 8.0 and analytical gel-filtration chromatography was performed in degassed and nitrogen bubbled 20 mM HEPES pH 7.5 plus 200 mM NaCl. The absorbance for Cu(I)-*Sl*Csp3 is divided by 15.0 in (B).

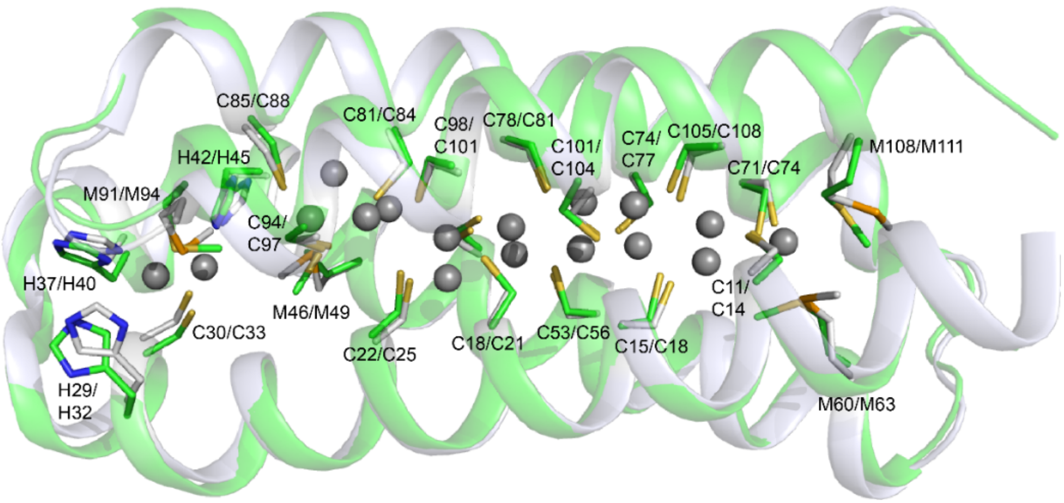


**Figure S9.** A comparison of the crystal structures of Cu(I)-*Rk*Csp3 and the apo-Csp3 from *Nitrosospira multiformis* ATCC 25196. An overlay [root mean square deviation (rmsd) ~0.8 Å for $C^{\alpha}$ atoms] of the crystal structures of Cu(I)-*Rk*Csp3 (chain A from 6ZIF in green) and the apo-form of the homologue from *N. multiformis* (*Nm*Csp3, pale blue, PBD code 3LMF), which belongs to the same sub-class. The Cu(I) sites are shown as grey spheres in Cu(I)-*Rk*Csp3 and the side chains of coordinating residues and His29, and the equivalent residues in apo-*Nm*Csp3 (all conserved) are show as sticks and are labelled (numbering: *Rk*Csp3/3LMF). For comparison, overlays of the Cu(I)-*Rk*Csp3 structure gives an rmsd (for $C^{\alpha}$ atoms) of ~1.4 Å with Cu(I)-*Mt*Csp3 (5ARN) and ~1.5 Å with Cu(I)-*Sl*Csp3 (6EK9). The rmsd (for $C^{\alpha}$ atoms) for the overlay of Cu(I)-*Mt*Csp3 (5ARN) and Cu(I)-*Sl*Csp3 (6EK9) is 0.41 Å.

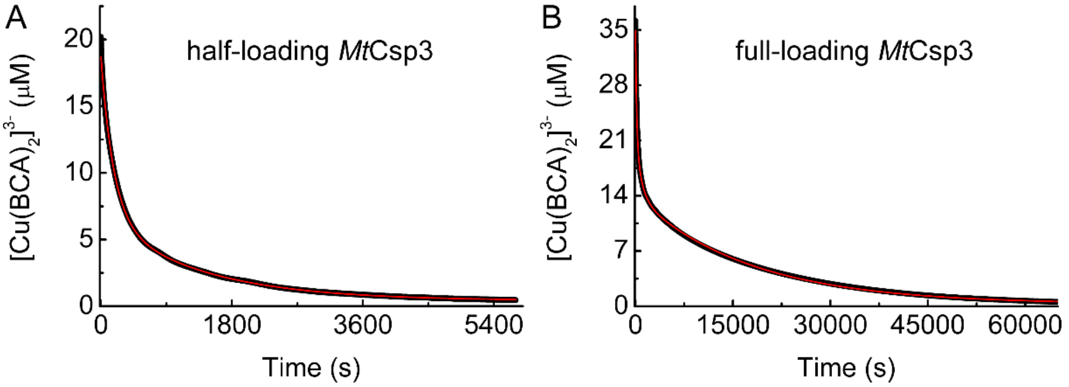


**Figure S10.** Fitting Cu(I) binding data for *Mt*Csp3. Plots of $[Cu(BCA)_2]^{3-}$ concentration against time (black lines) when adding 20.9 (A) and 36.4 (B) μM of the complex to apo-*Mt*Csp3 (2.22 μM). All reactions were carried out in 20 mM HEPES pH 7.5 plus 200 mM NaCl (22 °C), and monitored at 562 nm for up to 5700 and beyond 65000 s, respectively. The fit (red line) of the ~half-loading (9.8 equivalents) data (A) to two exponentials gives $t_1$ and $t_2$ values (decay rates) of $5.8 \times 10^{-3}$ $s^{-1}$ and $7.5 \times 10^{-4}$ $s^{-1}$, respectively. The ~full loading (17.1 equivalents) data have been fit to three exponentials giving $t_1$, $t_2$ and $t_3$ values of $5.4 \times 10^{-3}$ $s^{-1}$, $1.1 \times 10^{-3}$ $s^{-1}$ and $5.7 \times 10^{-5}$ $s^{-1}$, respectively. The average rates and standard deviations from replicates with all Csp3s are shown in Table S1.

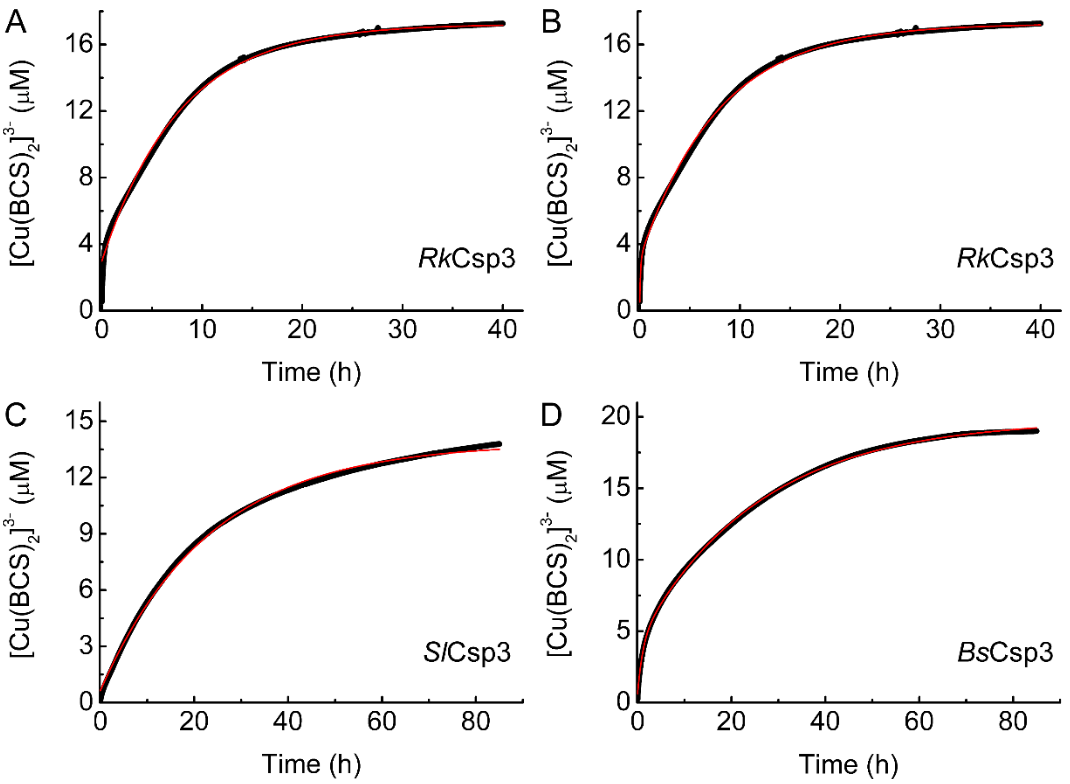


**Figure S11.** Fitting Cu(I) removal data for Csp3s. Plots of $[Cu(BCS)_2]^{3-}$ concentration against time (black lines) when adding BCS (2.4-2.5 mM) to Cu(I)-loaded *Rk*Csp3 (A and B), *Sl*Csp3 (C) and *Bs*Csp3 (D). *Rk*Csp3 (1.18 μM) was loaded with 16.1 equivalents of Cu(I) per monomer and a fit of the data (red line) to a single exponential (A) gave a removal rate of $1.3 \times 10^{-1}$ $h^{-1}$, whilst the fit to two exponentials (B) gave removal rates of 11.3 $h^{-1}$ (low amplitude phase) and $1.3 \times 10^{-1}$ $h^{-1}$ (majority of the reaction). The experiment with *Sl*Csp3 (C) used protein (1.11 μM) loaded with 18.1 equivalents of Cu(I) per monomer and the fit of the data (red line) to a single exponential gave a removal rate of $4.3 \times 10^{-2}$ $h^{-1}$. Fits are slightly better to two exponentials, but the rate obtained for the main reaction varies by up to ~6-fold for the three repeats. The fit shown (red line) to the data for *Bs*Csp3 (1.24 μM) loaded with 18.0 equivalents of Cu(I) per monomer is to two exponentials giving a removal rate of $3.9 \times 10^{-2}$ $h^{-1}$ (majority of the reaction). A fit to a single exponential gives a very similar rate ($4.2 \times 10^{-2}$ $h^{-1}$). All reactions were carried out in 20 mM HEPES pH 7.5 plus 200 mM NaCl (22 °C), and the average values and standard deviations from replicates with all Csp3s are shown in Table S2.

**Table S1. The Rates Obtained from Fitting the Cu(I) Binding Data for the Csp3s to Two (~10 eq. Data) and Three (~17-18 eq. Data) Exponentials.[a]**

| Protein | Cu(I) eq. added[b] | t1 ($s^{-1}$) | t2 ($s^{-1}$) | t3 ($s^{-1}$) |
|---|---|---|---|---|
| *Rk*Csp3 | ~10[c] | (1.1±0.2) × $10^{-1}$ | (1.7±0.4) × $10^{-2}$ | |
| | ~17[d] | (9.5±1.7) × $10^{-2}$ | (1.4±0.4) × $10^{-2}$ | (1.3±0.3) × $10^{-3}$ |
| *Mt*Csp3 | ~10[e] | (6.1±1.5) × $10^{-3}$ | (6.6±1.2) × $10^{-4}$ | |
| | ~18[f] | (5.6±1.7) × $10^{-3}$ | (7.0±2.7) × $10^{-4}$ | (4.4±0.9) × $10^{-5}$ |
| *Sl*Csp3[g] | ~10[c] | (7.6±1.2) × $10^{-2}$ | (1.4±0.3) × $10^{-2}$ | |
| | ~18[d] | (6.2±0.6) × $10^{-2}$ | (1.0±0.2) × $10^{-2}$ | (8.9±1.8) × $10^{-4}$ |
| *Bs*Csp3 | ~10[c] | (8.4±0.6) × $10^{-2}$ | (1.5±0.3) × $10^{-2}$ | |
| | ~18[d] | (8.9±1.4) × $10^{-2}$ | (1.6±0.4) × $10^{-2}$ | (1.3±0.2) × $10^{-3}$ |

[a]All reactions were carried out in 20 mM HEPES pH 7.5 plus 200 mM NaCl (22 °C). [b]Cu(I) added as the $[Cu(BCA)_2]^{3-}$ complex with equivalents ranging from: 9.6 to 10.4 eq. (n = 6) and 16.0 to 19.3 eq. (n = 7) for a *Rk*Csp3 monomer; 9.8 to 10.3 eq. (n = 5) and 17.1 to 18.3 eq. (n = 5) for a *Mt*Csp3 monomer; 10.1 to 10.5 eq. (n = 5) and 18.1 to 18.7 eq. (n = 5) for a *Sl*Csp3 monomer; 10.2 to 11.1 eq. (n = 6) and 18.5 to 18.9 eq. (n = 5) for a *Bs*Csp3 monomer. [c]Fits to two exponentials obtained using the initial 800 s of the reaction. [d]Fits to three exponentials obtained using the first 1800 s of the reaction. [e]Fits to two exponentials obtained using the first 5400-5700 s of the reaction. [f]Fits to three exponentials obtained using 63342-92131 s of the reaction. In some cases, fits of full-loading data for *Sl*Csp3 and *Bs*Csp3 to three exponentials gave slower rates (typically ~2-4-fold) when 5700 rather than only 1800 s of data were used. The majority of these reactions were completed by 1800 s and the $t_1$ and $t_2$ values obtained using this amount of data matched the values from fitting the half-loading data. Furthermore, fits using more data had a tendency to decrease, quite dramatically in some cases, the amplitude of the third phase of the reaction. [g]The approach used here is similar to that reported previously for studying the binding of Cu(I) to the initial sites occupied in *Sl*Csp3.[4] However, we have focused on reactions adding sufficient Cu(I) to approximately half and fully load the proteins.

**Table S2. The Rates Obtained for Cu(I) Removal from the Csp3s by BCS.**[a]

| Protein | Cu(I) equiv.[b] | Timescale of experiment (h)[c] | Removal rate ($h^{-1}$)[d] |
|---|---|---|---|
| *Rk*Csp3 | 15.9-17.1 (n = 5) | 40 | $(1.4\pm0.1) \times 10^{-1}$ |
| *Mt*Csp3 | 16.6-17.2 (n = 2) | 85 | $(1.8\pm0.4) \times 10^{-2}$ |
| | 16.6 (n = 1) | 615 | $1.5 \times 10^{-3}$ |
| *Sl*Csp3 | 15.8-18.1 (n = 3) | 85 | $(3.9\pm0.5) \times 10^{-2}$ |
| *Bs*Csp3 | 17.8-18.0 (n = 3) | 24-85 | $(3.8\pm0.1) \times 10^{-2}$ |

[a]The BCS concentration was ~2.4-2.5 mM and all reactions were carried out in 20 mM HEPES pH 7.5 plus 200 mM NaCl (22 °C). [b]The range of Cu(I) equivalents added per monomer prior to the removal experiments are listed with protein concentrations ranging from 1.08 to 1.26 μM. The number of repeats (n) are in parenthesis. [c]The time over which the formation of $[Cu(BCS)_2]^{3-}$ was monitored at 483 nm. [d]The rates quoted for *Rk*Csp3, *Mt*Csp3 and *Bs*Csp3 are for the main component from a fit of the data to two exponentials, whilst for *Sl*Csp3 all data fit reasonably well to single exponentials. Fits of the *Bs*Csp3 and *Rk*Csp3 data to a single exponential gave very similar rates for the majority of the reactions. Fits are slightly better to two exponentials for *Sl*Csp3, but the rate obtained for the main reaction varies ~6-fold for the three repeats.

**Table S3. Data Statistics and Refinement Details.**

| | *Rk*Csp3 (6ZIF) |
|---|---|
| **Data statistics[a]** | |
| Beamline | I24 |
| Date | 13/04/18 |
| Wavelength (Å) | 0.967 |
| Resolution (Å) | 47.77 – 2.20 (2.27 – 2.20 ) |
| Space group | $P4_32_12$ |
| Unit-cell parameters | |
| a (Å) | 60.76 |
| b (Å) | 60.76 |
| c (Å) | 231.93 |
| α = β = γ (°) | 90.00 |
| Unit-cell volume (Å$^3$) | 856233 |
| Solvent content (%) | 45 |
| No. of measured reflections | 269011 (24007) |
| No. of independent reflections | 22975 (1957) |
| Completeness (%) | 99.3 (100.0) |
| Redundancy | 11.7 (12.3) |
| $CC_{1/2}$ (%) | 0.997 (0.567) |
| <I>/<σ(I)> | 7.6 (1.6) |
| Anomalous completeness | 97.4 (99.9) |
| Anomalous redundancy | 6.1 (6.3) |
| | |
| **Refinement statistics** | |
| Rwork (%) | 21.07 |
| Rfree[b] (%) | 27.04 |
| No. of non-H atoms | |
| No. of protein, atoms | 3246 |
| No. of solvent atoms | 0 |
| No. of ligand atoms | 70 |
| R.m.s. deviation from ideal values | |
| Bond angle (°) | 1.69 |
| Bond length (Å) | 0.007 |
| Average B factor (Å$^2$) | |
| Protein | 49 |
| Solvent | N.A. |
| Ligand | 47 |
| Ramachandran plot[c], residues in | |
| Most favoured regions (%) | 98.34 |

[a]Values in parentheses are for the highest resolution shell. [b]5% of the randomly selected reflections excluded from refinement. [c]Calculated using Molprobity.[5]

## SUPPORTING REFERENCES

(1) Vita, N.; Landolfi, G.; Baslé, A.; Platsaki, S.; Lee, J.; Waldron, K. J.; Dennison, C. Bacterial cytosolic proteins with a high capacity for Cu(I) that protect against copper toxicity. *Sci. Rep.* **2016**, *6*, 39065.

(2) Straw, M. L.; Chaplin, A. K.; Hough, M. A.; Paps, J.; Bavro, V. N.; Wilson, M. T.; Vijgenboom, E.; Worrall, J. A. R. A cytosolic copper storage protein provides a second level of copper tolerance in *Streptomyces lividans*. *Metallomics* **2018**, *10*, 180–193.

(3) Notredame, C.; Higgins, D. G.; Heringa, J. T-Coffee: A novel method for fast and accurate multiple sequence alignment. *J. Mol. Biol.* **2000**, *302*, 205–217.

(4) Straw, M. L.; Hough, M. A.; Wilson, M. T.; Worrall, J. A. R. A histidine residue and a tetranuclear cuprous-thiolate cluster dominate the copper loading landscape of a copper storage protein from *Streptomyces lividans*. *Chem.-Eur. J.* **2019**, *25*, 1-12.

(5) Williams, C. J.; Headd, J. J.; Moriarty, N. W.; Prisant, M. G.; Videau, L. L.; Deis, L. N.; Verma, V.; Keedy, D. A.; Hintze, B. J.; Chen, V. B.; Jain, S.; Lewis, S. M.; Arendall, W. B.; Snoeyink, J.; Adams, P. D.; Lovell, S. C.; Richardson J. S.; Richardson, D. C. Molprobity: More and better reference data for improved all-atom structure validation. *Protein Sci.* **2018**, *27*, 293–315.
